# Toward an open‐source 3D‐printable laboratory

**DOI:** 10.1002/aps3.11562

**Published:** 2024-01-18

**Authors:** Mason C. McNair, Sebastian C. Cocioba, Peter Pietrzyk, Trevor W. Rife

**Affiliations:** ^1^ Plant and Environmental Sciences, Pee Dee Research and Education Center Clemson University 2200 Pocket Road Florence South Carolina 29506 USA; ^2^ Binomica Labs 4301 22nd Street, Floor 3, Studio 342, Long Island City New York 11101 USA; ^3^ Department of Plant Biology University of Georgia 120 Carlton Street Athens Georgia 30602 USA

**Keywords:** 3D printing, affordable, citizen science, low‐cost, open‐source, wet lab

## Abstract

**Premise:**

Low‐cost, repairable lab equipment is rare within the biological sciences. By lowering the costs of entry using 3D printing and open‐source hardware, our goal is to empower both amateur and professional scientists to conduct research.

**Methods:**

We developed a modular system of 3D‐printable designs called COBLE (Collection of Bespoke Laboratory Equipment), including novel and remixed 3D‐printable lab equipment that can be inexpensively printed, assembled, and repaired for a fraction of the cost of retail equivalents.

**Results:**

Here we present novel tools that utilize 3D printing to enable a wide range of scientific experiments. We include additional resources for scientists and labs that are interested in utilizing 3D printing for their research.

**Discussion:**

By describing the broad potential that 3D‐printed designs can have in the biological sciences, we hope to inspire others to implement and improve upon these designs, improving accessibility and enabling science for all.

Scientific progress is limited by the accessibility and availability of lab and research equipment. To enable science for all, easily obtainable scientific equipment is vital to researchers both inside and outside academia (Nosek et al., 2015; Price‐Whelan et al., 2018; Popkin, 2019). However, many research labs operate with restricted budgets that limit the purchase of new hardware and are often forced to creatively reuse inherited, outdated, or damaged hardware to perform research. Recent technical advancements, affordability, and accessibility of 3D printers have allowed resource‐restricted researchers to become makers, manufacturing the low‐cost hardware needed for their specific research workflows. These custom‐printed solutions can be produced at a fraction of the cost of retail alternatives and can be rapidly designed and fabricated, avoiding potential supply chain issues that often affect research equipment (Del Rosario et al., 2021). Numerous do‐it‐yourself (DIY) scientific equipment designs are openly available on the internet, including pipettes, microscopes, centrifuges, and 3D scanners (Appendix S1). These existing designs can be further “remixed” to answer specific research questions or retrofit existing equipment (Jones et al., 2011; Cressey, 2017; Kwok, 2017; García‐Rojas et al., 2022).

This transformative shift toward open‐source hardware has resulted in the development of affordable tools and technologies across broad research and educational domains (Dawood et al., 2015; He et al., 2016; Yan et al., 2018; Ford and Minshall, 2019; Hansen et al., 2020; Del Rosario et al., 2021). The addition of a consumer‐grade 3D printer to a research laboratory has the potential to save thousands of dollars in lab equipment purchases. Like other common lab equipment, 3D printers require time to manage and maintain; however, they afford labs the opportunity to rapidly evaluate and implement custom hardware and tools with limited reliance on external vendors. The growing community of open‐source model designers is making it possible to perform research with lab‐specific, highly customized hardware that caters to the needs of researchers on an individual level.

For the biological sciences, 3D printing can be used to create new equipment, replicate equipment that would otherwise be prohibitively expensive, or customize existing models (Figure 1). Although there may be a perception that 3D printing has high barriers to entry such as cost and the requirement for specialized knowledge, these obstacles can be overcome with minimal time, effort, and a basic understanding of 3D printing (Figure 1). Scientists can evaluate the potential of 3D printing for their own lab equipment by working with public makerspaces and printing services (Grace‐Flood, 2017; All3DP, 2023). These groups often hold workshops and training sessions that can help guide researchers through the process of purchasing and using their own printer. Because of these perceived barriers, the adoption of 3D printing technology has been slow, but within the plant sciences multiple projects already implement 3D printing as part of their workflow. From entire systems enabling non‐destructive root and shoot imaging; tissue culture vessels; models of roots, shoots, and pollen; and bio‐printed functional plant cells, these 3D‐printed projects are wide reaching. They have applications in hydroponics and tissue culture (Mathieu et al., 2015; Shukla et al., 2017), root system architecture (Liang et al., 2017; Arnaud et al., 2019), phenomics (Griffiths, 2020), education and outreach (Perry et al., 2017; Wilson, 2023), and more (Gao et al., 2018; Sasse et al., 2019; Mehrotra et al., 2020; Van den Broeck et al., 2022).

**Figure 1:**
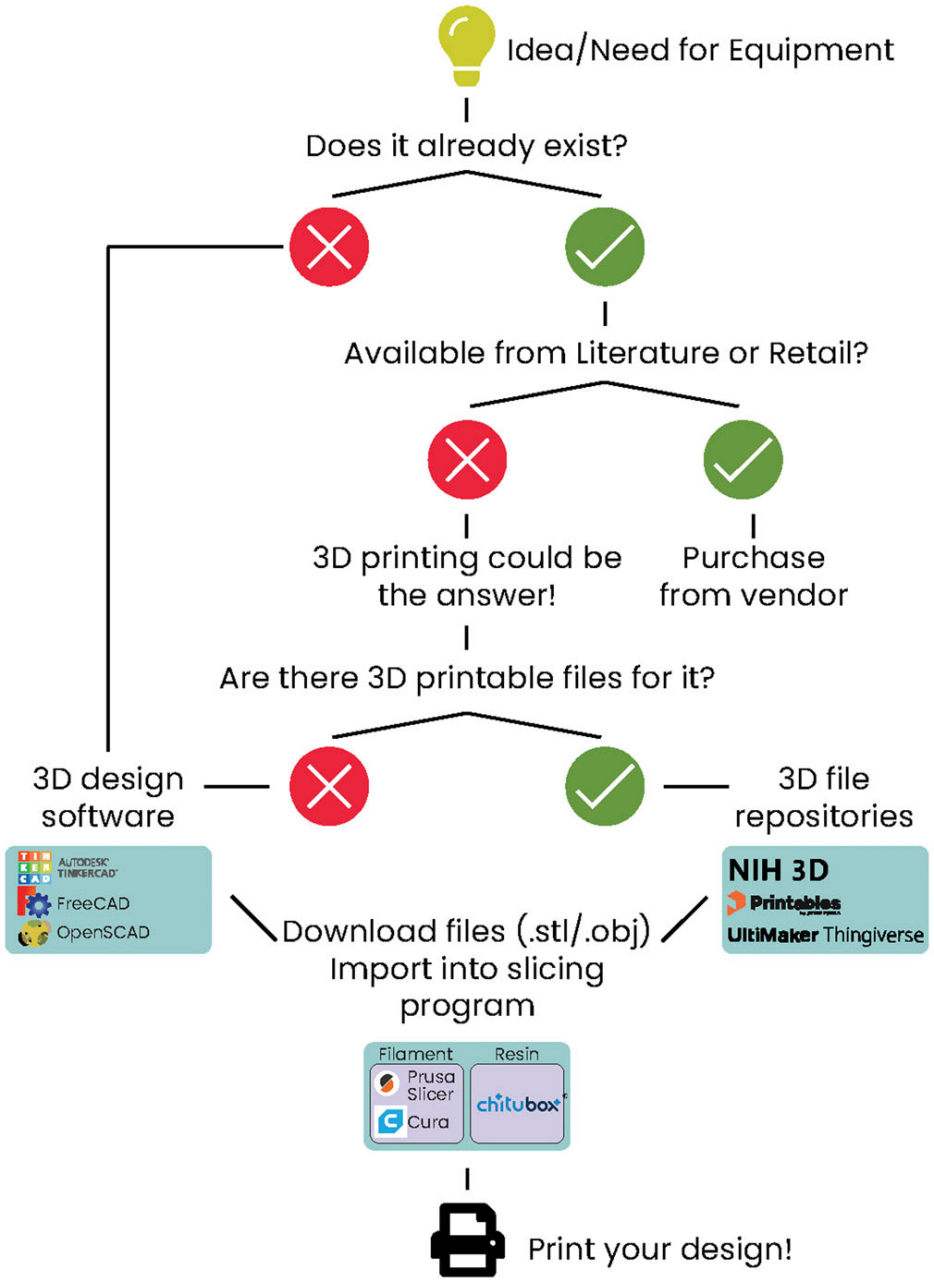
Flowchart showing considerations in deciding whether to 3D print and three popular free resources for file availability, 3D design software, and slicing programs.

The technology central to most 3D printing, fused filament fabrication (FFF), uses layered molten filament to render 3D structures out of 2D planar segments and has advanced significantly over the past decade such that the central concern of dimensional tolerances between printers has been all but eliminated (Jones et al., 2011; Ning et al., 2015; Shaqour et al., 2021). An alternative technology that is rising in popularity is stereolithography (SLA) printing, which uses a liquid UV‐curable resin and a high‐resolution liquid crystal display (LCD) screen or digital light processing (DLP) projector to render each layer as a photolithographic mask. This technology allows for micron‐resolution, which enables researchers to render fine structures like microfluidic systems, a field that has been limited to resource‐rich labs due to high material costs (Macdonald et al., 2017). While SLA printing has strong potential for laboratory applications, the high‐resolution output is not typically needed for most DIY laboratory equipment. To navigate the rising complexity in the field of 3D printing, we have created a simplified guide that outlines the current landscape of printing technologies and includes suggestions for adoptable 3D printers (Figure 2) based on similar guides found on popular 3D printing forums (e.g., u/richie225, 2022; All3DP, 2023; Simplify 3D, 2023).

**Figure 2:**
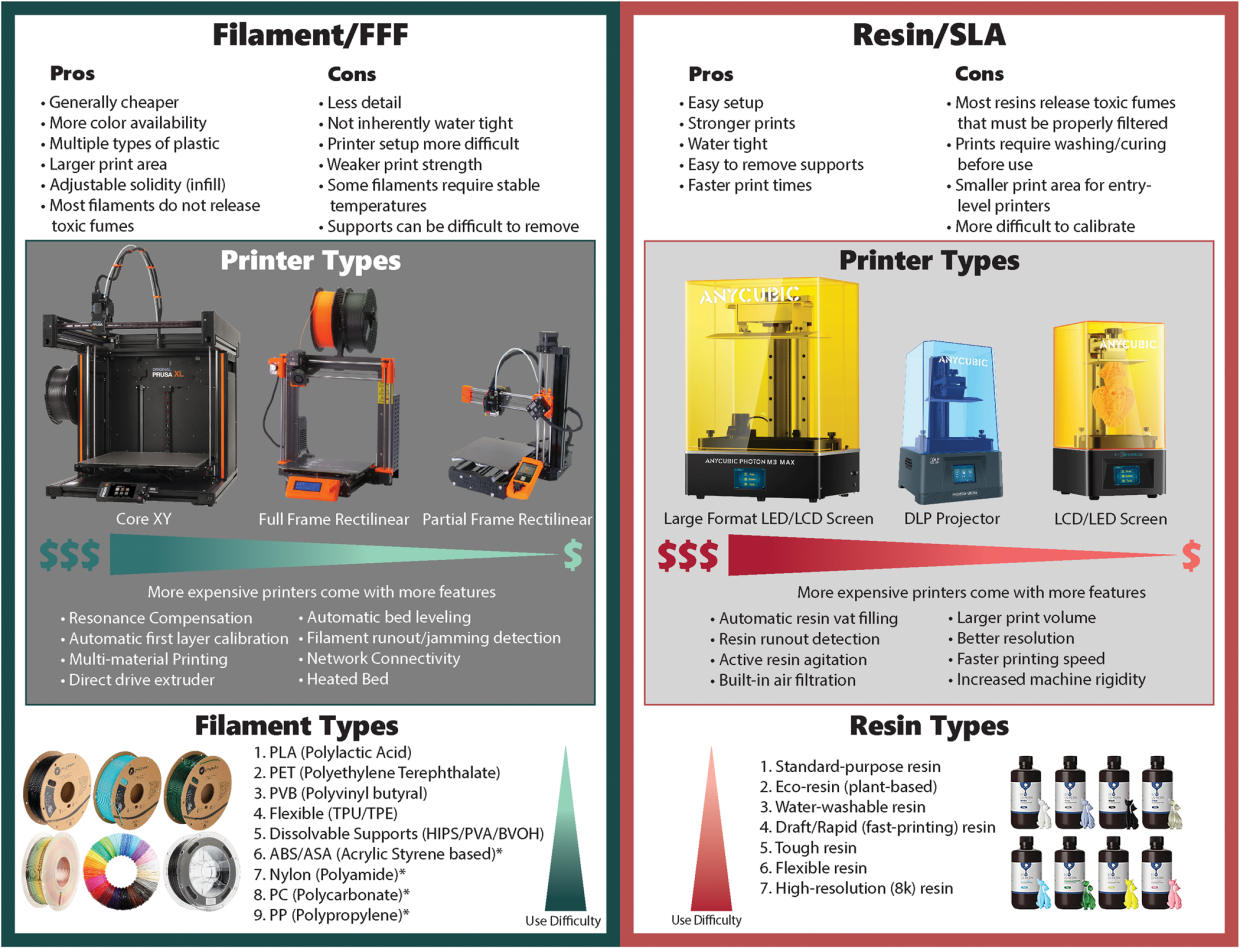
Buying guide for entry‐level 3D printers, showing pros and cons for each type of 3D printer, popular options for printer types (fused filament fabrication [FFF] and stereolithography [SLA]), and reliable filament/resin types on the market at the time of publication. Lists of printer, filament, and resin types are not exhaustive as there are many brands of printers and materials available. The authors prefer Prusa Research (Prague, Czech Republic) printers for FFF printing and Anycubic (Shenzhen, China) printers for SLA printing.

Even with the increased accessibility, interest, and publications utilizing 3D printing for research, general adoption in the biological sciences remains low. For inexperienced users, finding the appropriate equipment for specific needs can be tedious and time consuming. This is exacerbated by the lack of a stable, central, public repository for model designs and instructions. Printable files can be accessed through a variety of online repositories, each with their own interface and community. Three popular repositories include Prusa Research's Printables (https://www.printables.com/), UltiMaker's Thingiverse (https://www.thingiverse.com/), and NIH 3D (https://3d.nih.gov/). Printables and Thingiverse are maintained by their private parent companies and have the largest collection of 3D‐printable designs. NIH 3D is maintained by the National Institutes of Health, allowing it to be a more reliable long‐term archive for 3D‐printed designs, but it is generally less user‐friendly than the other repositories. Few publications that utilize 3D printing for research ultimately upload their designs to these repositories, resulting in designs hosted with the publishing journal, on code repositories like GitHub, or on lab websites. These designs are nearly impossible to find through traditional web searches, raising a key consideration: If 3D‐printed tools are not able to be found, customized, and easily manufactured, the intended purpose of these tools becomes lost.

To demonstrate the importance of organizing, validating, and openly sharing related 3D‐printable designs, we have created COBLE (Collection of Bespoke Laboratory Equipment), a group of designs that incorporates models from both peer‐reviewed publications and publicly accessible repositories. COBLE is a modular system of 3D‐printable designs created or remixed by the authors to facilitate research in biological wet labs and includes the Biological Affordable Imaging with Raspberry Pi (BAIR) and BeadMag systems. The system targets common wet lab activities including DNA extraction, quality assurance, and library preparation, but the individual tools can be easily adapted for myriad uses. Here, we provide the information needed to print your own COBLE system, along with numerous other pieces of original and remixed 3D‐printed lab equipment. These designs have been tested in both home and academic plant biology and plant pathology laboratory settings.

## METHODS AND RESULTS

**Figure 3:**
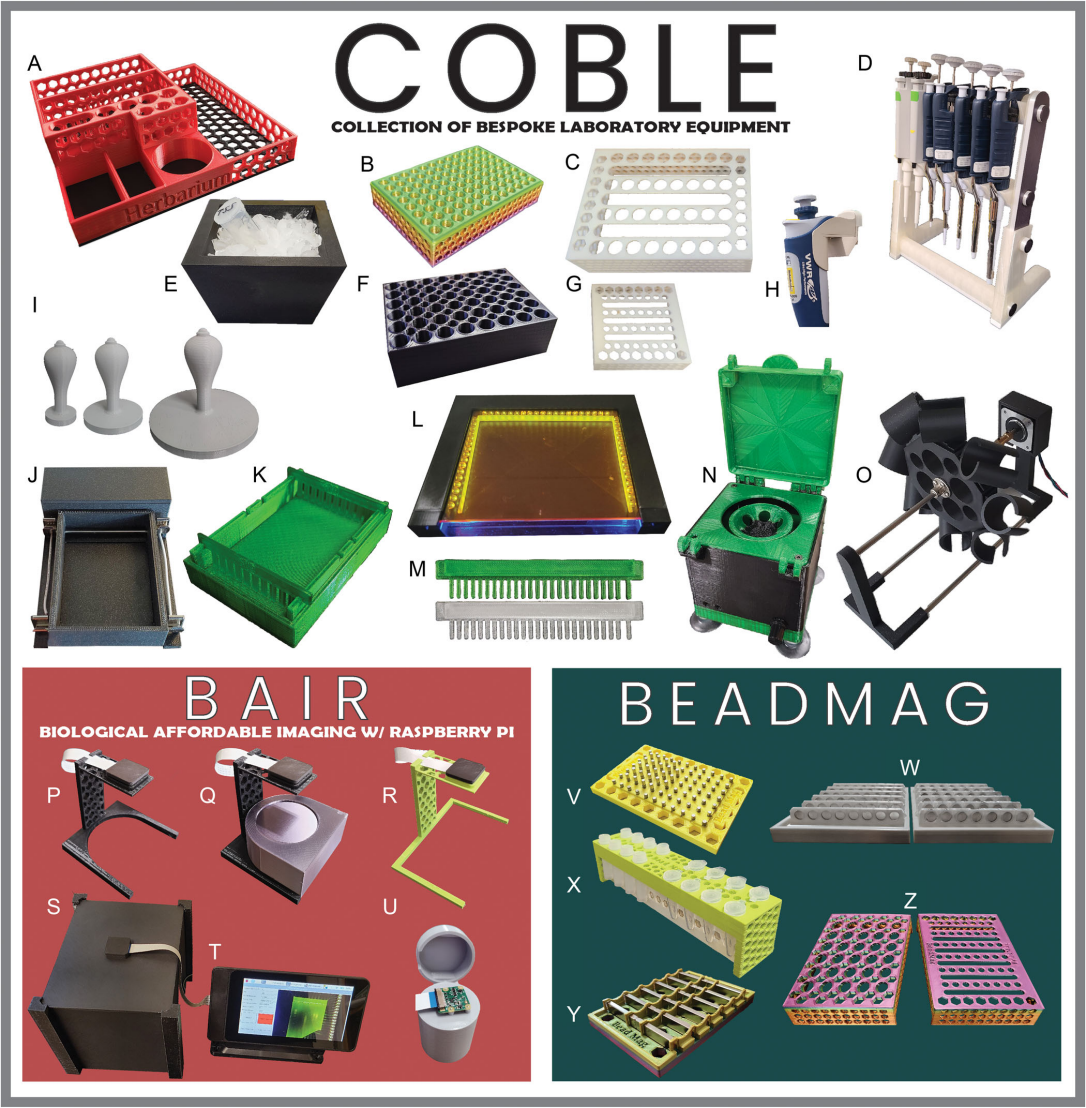
Samples of all COBLE system equipment. (A) Herbariorganizer tool storage. (B) 0.2‐mL tube stackable freezer storage block. (C) 1.5/2‐mL tube rack. (D) Pipette stand. (E) Ice bucket. (F) Geno/Grinder 1.5/2‐mL tube block. (G) 0.2‐mL tube rack. (H) VWR multichannel pipette shelf adapter. (I) Filter paper tamps (50–150 mm). (J) Gel electrophoresis unit. (K) Gel casting rig and combs. (L) Transilluminator. (M) Owl EasyCast Replacement Combs. (N) DIY mini adjustable speed centrifuge. (O) Sir Tumbalot tube tumbler. (P) BAIR 100‐mm Petri dish camera adapter. (Q) BAIR 60‐mm Petri dish camera adapter. (R) BAIR 96‐well plate camera adapter. (S) BAIR transilluminator hood. (T) BAIR system. (U) BAIR microscope adapter. (V) BeadMag microtiter magnetic separator. (W) BeadMag plate magnetic separator (round magnets version). (X) BeadMag 1.5/2‐mL tube magnetic separator. (Y) BeadMag plate magnetic separator (rectangular magnet version). (Z) BeadMag plate magnetic separator tube adapters (1.5 mL and PCR strip tubes).

We compiled an extensive list of uploaded work from other makers comprising designs carefully selected for ease of printing, robustness, and community feedback via ratings and comments. Within this collection, we identified designs that were able to replace or extend the most commonly used wet lab benchtop hardware for cetyltrimethylammonium bromide (CTAB)–based DNA extraction. We developed new hardware and remixed existing designs before testing using established laboratory protocols (Appendix S2). COBLE currently contains files to create a tabletop centrifuge, gel electrophoresis system, agarose gel casting tray, gel combs (Thermo Fisher Scientific, Waltham, Massachusetts, USA), ice bucket, Petri dish tamps, tissue grinder tube blocks (Geno/Grinder; Cole‐Parmer, Metuchen, New Jersey, USA), stackable freezer tube storage blocks, magnetic separator blocks, tube racks, a transilluminator, and the BAIR imaging system (Table 1, Figure 3). Instructions for assembly, bill of materials, and recommended non‐printed part sources are provided in Appendix S3. Although this collection does utilize some non‐3D‐printed parts that may have varied availability (Chowdhury et al., 2021), the cost and accessibility of these materials were considered throughout the design and remixing process.

**Table 1:** Cost comparison of 3D‐printed equipment to retail equivalents. All items listed were developed by the authors.

Equipment	Figure 3 image	3D‐printed item^a^	Printed cost (2023 USD)^b^	Retail cost (2023 USD)^b^
COBLE: Collection of Bespoke Laboratory Equipment	A	Herbariorganizer herbarium tool storage	6	—
	B	Stackable freezer storage block	4	22
	C and G	Tube racks	2	16
	D	Pipette stand	13	145
	E	Ice bucket	12	128
	F	Geno/Grinder (Cole‐Parmer, Metuchen, New Jersey, USA) tube block	6	373
	H	VWR (Avantor, Radnor, Pennsylvania, USA) multichannel and electric pipette shelf adapters	4	—
	I	Filter paper tamps	5	—
	J	Gel electrophoresis unit	25	659
	K	Gel casting rig and combs	30	659
	L	Transilluminator (for gel green or equivalent)	36	583
	M	Owl EasyCast (Thermo Fisher Scientific, Waltham, Massachusetts, USA) replacement combs	1	155
	N	DIY mini adjustable speed centrifuge	36	1000
	O	Sir Tumbalot tube tumbler	144	434
BAIR: Biological Affordable Imaging with Raspberry Pi	P	BAIR 100‐mm plate camera adapter	1	—
	Q	BAIR 60‐mm plate camera adapter	1	—
	R	BAIR 96‐well plate camera adapter	1	—
	S	BAIR Dark Box with Pi camera attachment	14	147
	T	BAIR imaging system	235	1600
	U	BAIR microscope adapters	2	66
		BAIR All	254	1830
BeadMag magnetic separators and tube adapters	V	BeadMag microtiter separator	14	940
	W and Y	BeadMag 96‐well plate separator	10	931
	X	BeadMag 1.5/2‐mL tube separator	10	665
	Z	BeadMag 1.5‐mL and PCR strip tube adapters	2	—

^a^ 3D‐printed equipment assumes 40% infill, includes supports when necessary, and includes additional purchased hardware in cost estimates.

^b^ Costs are rounded up to the nearest whole dollar. Retail costs are based on listed prices from major scientific equipment suppliers (e.g., Fisher Scientific, Thomas Scientific, Carolina Biological).

**Figure 4:**
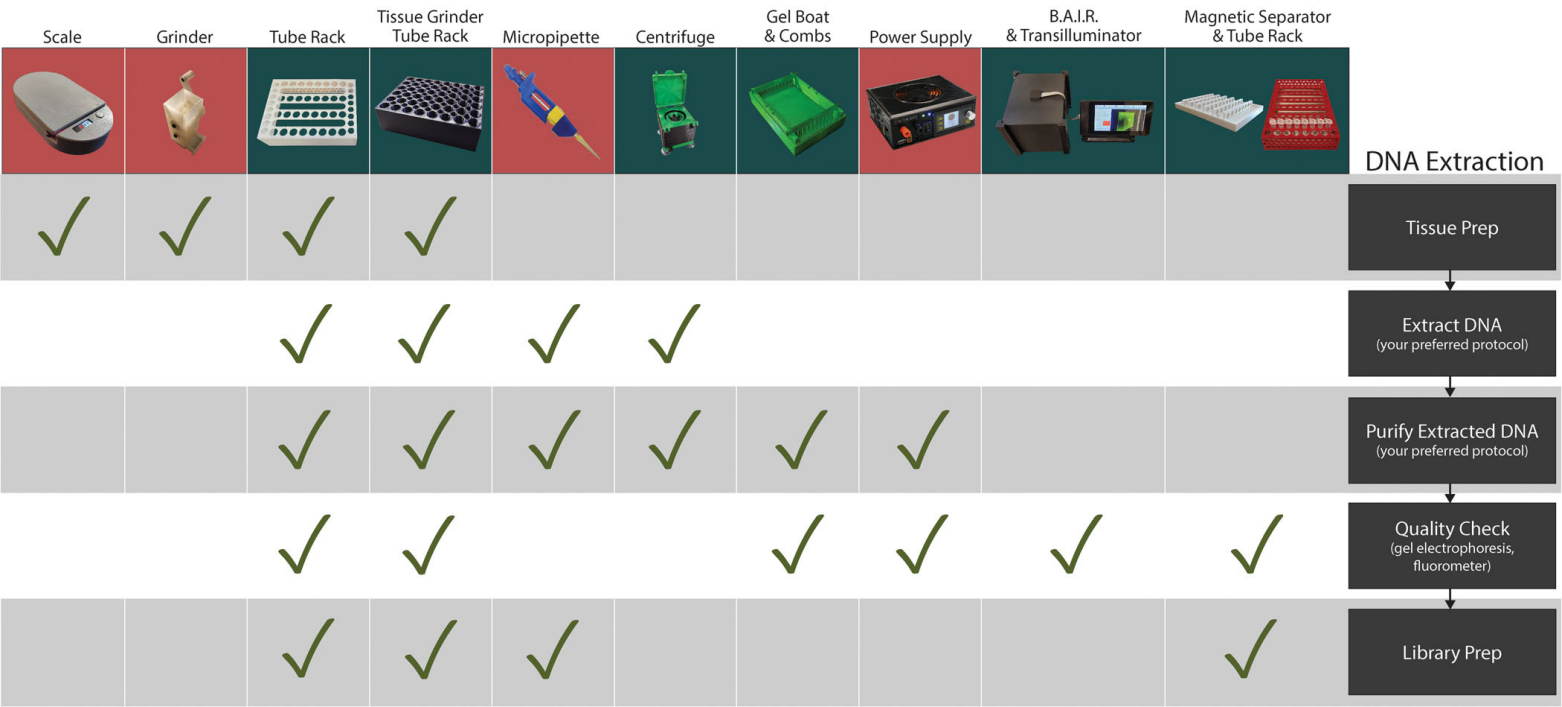
3D‐printed lab equipment and the steps of a typical DNA extraction when they can be used. Devices shown with green backgrounds were created by the authors and those with red backgrounds were found in online design repositories. Designs for tissue preparation included a Bluetooth‐enabled scale by “Valentin B” (Printables #36112), a hand‐cranked tissue grinder designed by “rmcht” (Thingiverse #2654215), and modular tube racks and blocks for Geno/Grinders designed by the authors alongside a printable ice bucket (Printables #45750). DNA extraction and purification employed the modular tube racks, tabletop centrifuge (remixed), gel electrophoresis rigs, BeadMag magnetic separators, and the BAIR system designed by the authors as well as biropettes by “BadenLab” (Thingiverse #255519), and a benchtop power supply by “KRALYN3D” (Thingiverse #3831773). DNA library preparation utilized the modular tube racks, BeadMag magnetic separators, and BAIR system by the authors, and biropettes by “BadenLab” (Thingiverse #255519).

While CTAB‐based DNA extraction protocols can vary considerably between applications, they all generally include steps requiring sample tissue breakdown (e.g., grinding), incubation, centrifugation, and pipetting (Doyle and Doyle, 1987). Following a modified protocol used in the Leebens‐Mack lab at the University of Georgia, we evaluated the efficacy of freely available designs, custom remixed designs, and entirely novel designs during extraction of more than 400 samples from flash‐frozen, silica‐dried, and pressed herbarium specimen tissues (Appendix S2). The most cost‐effective and mechanically robust designs are included with a comparison to commercial equivalents (Table 1, Appendix S4) and in our outline of the DNA extraction process (Figure 4). Quality control is the final step in most genomic DNA extraction methods. DNA agarose gel electrophoresis is commonly used for assessing quality and quantity, but all‐in‐one gel imaging systems can be a significant expense. To increase access and standardize this process, we developed the BAIR system, which facilitates the imaging of DNA agarose gels and can be adapted to other research imaging applications.

**Figure 5:**
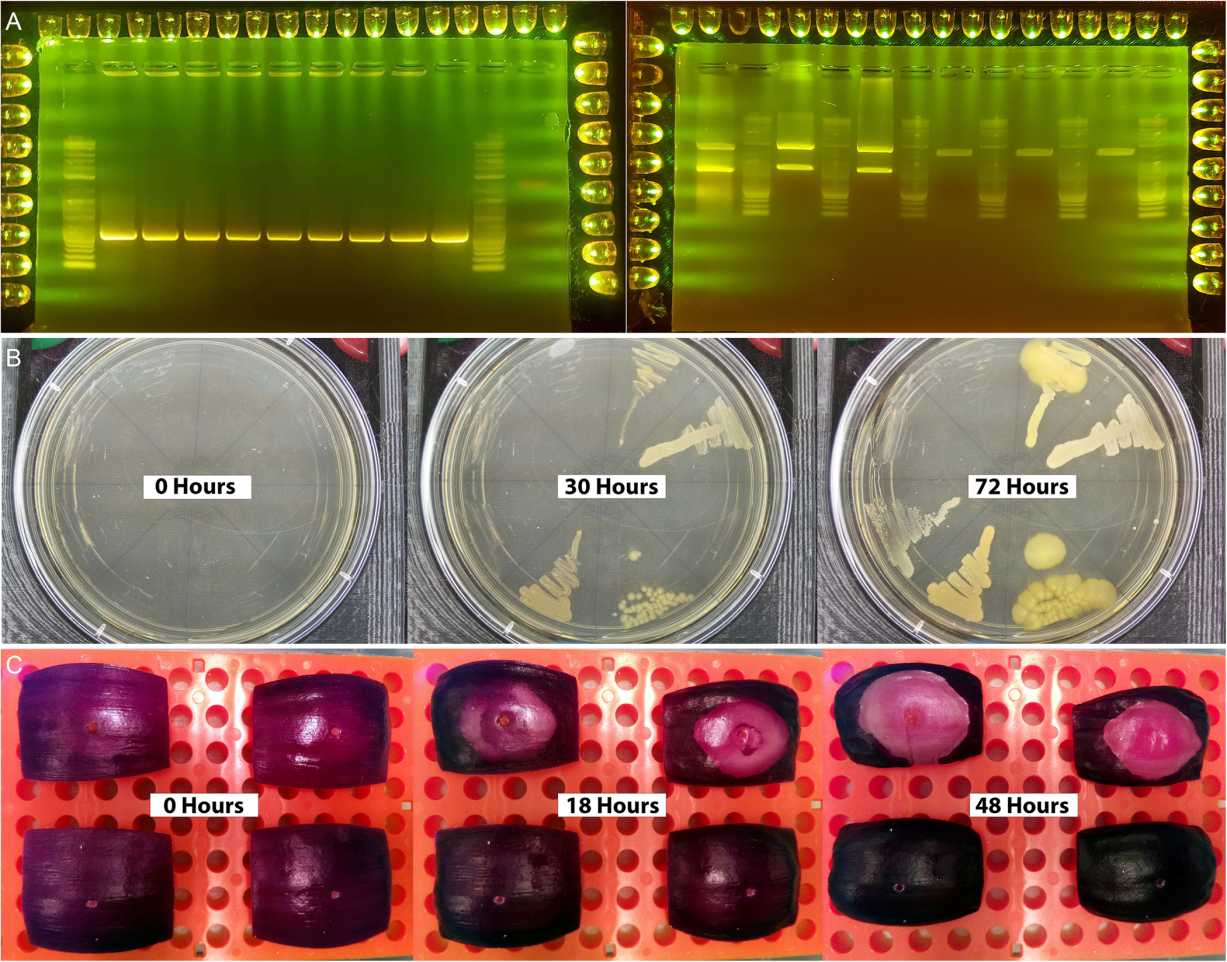
Potential imaging applications of BAIR. (A) Images of gel electrophoresis analyses with NEB 1KB Plus Ladder (New England Biolabs, Ipswich, Massachusetts, USA) taken using a cell phone camera (Left: Colony PCR with positive control on rightmost lane. Expected size around 500 bp. Right: Restriction digest and cloning of golden braid level zero parts into entry vectors. Cut by Esp3I). (B) Growth rates and phenotype of multiple *Curtobacterium* spp. in 100‐mm round plates. (C) Infection spread and patterning of *Pantoaea ananatis* in red onion scales. Full time‐lapse videos are provided in Video S1.

The BAIR system combines a Raspberry Pi computer and camera module (https://www.raspberrypi.com; Raspberry Pi Foundation, Cambridge, United Kingdom) with 3D‐printed microscope, plate imaging, and gel imaging attachments (Figure 3). It is controlled through a simple graphical user interface (GUI) developed in Python that allows users to adjust relevant camera settings including exposure/shutter speed, resolution, and ISO (International Organization for Standardization; commonly defined as film sensitivity). The interface also includes an intervalometer, which can be used to create time‐lapse video. Users have the option to control the time between images and limit the time lapse by either total time or number of images (Figure 5, Video S1). BAIR is open source, allowing other labs to adapt it to their specific needs. Opportunities for future feature development include enabling autofocus, high‐resolution imaging, and night‐vision cameras.

To validate the potential of COBLE, we have compared these tools to equivalent commercially available laboratory hardware. These inexpensive hardware designs have been successfully used in formal laboratory settings with satisfactory results. Precision for printed devices varies depending on the print quality and hardware used; however, the centrifuge has an approximate maximum output of 9660 relative centrifugal force (rcf) at 12,000 rpm and the biropette has an approximate 2.5% error at 200 μL. COBLE designs are extremely cost‐effective compared to commercial lab equipment, costing on average only 8.18% as much as the equivalent commercial hardware (Table 1). Furthermore, these solutions can be readily fixed, modified, and upgraded with improved designs or replacement hardware.

## DISCUSSION

The COBLE system and the additional equipment developed by other makers collated here allow anyone with access to a 3D printer to complete a CTAB‐based DNA isolation, bead‐based cleanup, library preparation, and quality check of their samples (Figure 4). Given the nature of these open‐source tools, these designs can be easily modified to fit specific needs, enhance functionality, or remixed into completely new devices that serve niche but necessary purposes (e.g., change an existing design for a plate holder to fit a differently sized plate). Designing with modularity and adaptability as the primary focus allows researchers to develop tools that can be used across diverse academic and educational environments (Zengler et al., 2019; Collins et al., 2020; McEvoy, 2023). The ability to develop and share new and remixed designs with remote partners allows for the rapid evaluation and adoption of custom laboratory and research equipment. This new paradigm has allowed researchers to bypass many commercial laboratory hardware suppliers where high prices act as a barrier to discovery and interruptions to distribution can cause significant delays to time‐sensitive research.

The free and rapid exchange of design files lowers the financial barrier of entry to biological research, especially in labs in developing countries or remote locations where rapid and efficient bootstrapping of a functional modern research laboratory is limited by equipment availability (Jones et al., 2011; Ning et al., 2015; Shaqour et al., 2021). Educators benefit the most from this open‐source approach. Prices for educational materials and supplies are often extraordinarily high (Walker, 2018; Ford and Minshall, 2019). The ability to customize and 3D print tools for classroom‐based research will allow students to experience and develop the methods and critical thinking needed to conduct basic research. These lessons will influence the career decisions of future scientists; the sooner a student becomes exposed to wet lab biology, the more capable they will be when they begin to engage in formal research (Kennedy and Odell, 2014; Aladé et al., 2016; de Philippis, 2021).

As 3D printing technology advances, higher‐resolution prints will become available, further lowering the barriers to more complex areas of research that have been previously reserved for labs with more resources. For example, in microfluidics, researchers typically pattern photolithographic surfaces to the order of single microns using light‐sensitive resists that are then used to form molds for casting polydimethylsiloxane (PDMS) elastomer slabs (Raj M and Chakraborty, 2020). These slabs are sandwiched onto glass plates to form flowable channels. Previous approaches for this process required expensive silicon wafers used in advanced electronics manufacturing, dramatically increasing the technical and financial barriers to entry (Iliescu et al., 2012). However, existing SLA technologies are able to render micron‐resolution positive reliefs of microfluidic circuits at minimal cost (Macdonald et al., 2017). With further refinement of both resin monomers and digital light masking technology, the resolution of future 3D‐printed microfluidic systems will increase in both quality and, more importantly, accessibility (Bhattacharjee et al., 2016; Nielsen et al., 2020).

With regards to model design, open‐source and low‐cost platforms are widely available to streamline adoption of these novel tools by allowing researchers to modify, enhance, and distribute new designs. Numerous tutorials are freely available for all major computer‐aided design (CAD) platforms, including some cloud‐based platforms that enable simultaneous design by multiple users. The collaborative nature of these platforms (e.g., Tinkercad [https://www.tinkercad.com/], KiCad [https://www.kicad.org/], FreeCAD [https://www.freecad.org/], and openSCAD [https://openscad.org/]) has saved researchers enough time and money to justify further inquiry into building toward a more accessible research landscape (Pearce, 2020). Their intuitive interface and robust user communities enable novices in 3D design, electrical engineering, coding, and programming to develop exciting software and hardware to further enhance the capabilities of what is presented in this paper and beyond.

Once a design has been finalized, we urge the scientific community to upload it to a highly trafficked public repository. Making new designs publicly accessible enables scientists with similar research questions to readily implement the design into their own laboratory protocols. Further downstream, a single design may serve as inspiration for many scientists with entirely different research questions. Public repositories often allow interactions between designer and user, providing an opportunity for feedback to flow upstream and further improve designs. These repositories also allow users to link remixed designs to the original, allowing future users to track modifications as they are created.

To encourage the adoption of these tools in the biological sciences, we compiled a list of potential lab equipment designs across the entirety of the NIH 3D, Printables, and Thingiverse repositories (Appendix S1), as well as some of the most useful guides to 3D printing that can be utilized by those with any experience level (Appendix S3). There is no better time to adopt 3D printing into your research lab. We hope that the advances in affordable equipment outlined in this paper inspire others to improve upon our designs and create more 3D‐printed innovations.

## AUTHOR CONTRIBUTIONS

M.C.M. and S.S.C. conceived the research, validated results and hardware robustness, provided resources for testing, and wrote the manuscript. T.W.R. aided in writing and conceptualization revisions. P.P. wrote all software and relevant portions of the manuscript associated with software. All methodology, data curation, visualization, supervision, and project administration was completed by M.C.M. All authors approved the final version of the manuscript.

### Open Research Badges

This article has earned Open Data and Open Materials badges for making publicly available the digitally shareable data necessary to reproduce the reported results. Data and materials are available at https://www.printables.com/model/45750.

## Supporting information


**Appendix S1**. 3D‐printable lab equipment found on common 3D print design repositories.

**Appendix S2**. Modified CTAB DNA extraction and library preparation protocols used to test equipment fabricated for COBLE.

**Appendix S3**. Step‐by‐step guide to assembly of all COBLE equipment listed in Table 1.

**Appendix S4**. 3D‐printable lab equipment found on common 3D print design repositories used by the authors during validation of the COBLE system.

**Video S1**. Time‐lapse videos made using the BAIR imaging system. (00:00 s) *Curtobacterium* spp.; (00.11 s) *Geranium maculatum* (spotted geranium) blooming; (00:27 s) *Sarracenia* seed germination experiencing drought; (00:43 s) *Gossypium* sprouting; (01:12 s) *Pantoea ananatis* infections on red onion slices; (01:31 s) *Cornus florida* flowers opening.

## Data Availability

All 3D designs and related data are freely available via Printables.com (https://www.printables.com/model/45750-coble-collection-of-bespoke-laboratory-equipment/) under a Creative Commons CC‐BY‐SA license. The BAIR GUI is freely available on https://github.com/PeterPieGH/BAIR. Should you need customization of any designs or access to a 3D printer, the corresponding authors may be able to help.
