# Supplementary material for: Toward an open‐source 3D‐printable laboratory
Source: Appl Plant Sci. 2024 Jan 18;12(1):e11562. doi: 10.1002/aps3.11562 (PMC10873812; doi:10.1002/aps3.11562)
Supplement: Supplementary file 2 — Appendix S2. Modified CTAB DNA extraction and library preparation protocols used to test equipment fabricated for COBLE. [file APS3-12-e11562-s003.docx]

**Appendix S2.** Modified CTAB DNA extraction and library preparation protocols used to test equipment fabricated using COBLE.

**CTAB *Cercis* Extraction (modified from Doyle and Doyle, 1978)**

Recipes (for 100 extractions):

100 mL CTAB

- 10 mL Tris
- 4 mL 0.5M EDTA
- 8.181 g NaCl
- 2 g CTAB
- 2 g PVP
- H_2_O to volume

100 mL TE (Light)

- 98.8 mL distilled H_2_O
- 1 mL 1 M Tris HCl pH 8.0
- 0.2 mL 0.5 M EDTA

100 mL Chloroform Isoamyl Wash

- 96mL Chloroform
- 4mL isoamyl alcohol

**Day 1**

1. Label one 2-mL and three 1.5-mL microcentrifuge tubes on the top and side for each sample DNA will be extracted from.
2. Set the heat block to 65°C.

NOTE: Steps vary from here to step 10 (adding 2-mercaptoethanol [BME]). Depending on taxon for dry tissue, it may be advisable to add CTAB prior to grinding. (If yields are lower than expected, adding an additional 10–15% PVP prior to the BME may be advisable.)

***Cercis* leaf and bud tissue (frozen)**

1. Add two ceramic beads to the labeled 2-mL tube.
2. Add sample material (0.1 g) to the 2-mL tube.
3. Add one steel bead to the 2-mL tube.
4. Freeze the 2-mL tubes in liquid NO_2_ and take them to the beadmill.
5. Run samples in the beadmill at speed 4 m/s for 30 s, allow them to cool for 1 min, then repeat for a total of two rounds of grinding; all samples should be ground into a fine powder. If this is not the case, repeat for an additional 30 s with 1 min of cooldown until sample appears as fine powder (likely for buds).
6. Add 600 μL 2% CTAB buffer to each tube and vortex until all beads are loose (can be heard moving during vortexing).
7. Centrifuge all samples briefly (speed 18000 rcf for 15–20 s then press stop) to pull all material to the bottom of the tube.
8. IN FUME HOOD: Add 200 μL 99% 2-mercaptoethanol (BME) to each sample tube. Mix the contents to the tube through vortexing.
9. Incubate at 65°C for 30 min. Mix each sample through vortexing every 10 min.
10. After 30 min, samples can be stored at −20°C, or you can continue on to step 14.

Stopping Point (or Continue)

1. If the samples were stored at −20°C, place them on a 65°C heat block for 15 min. Mix each sample through vortexing after 10 min.
2. Centrifuge samples for 3 min at 18,000 rcf.
3. Transfer the supernatant into a new labeled tube without collecting any solid particles (P200 micropipette).
4. Add an equivalent volume of 24:1 chloroform:isoamyl alcohol to each tube and mix by hand for 30 s (solution should appear cloudy).
5. Centrifuge for 3 min at 18000 rcf.
6. Carefully transfer the aqueous phase (generally the top layer) into a new labeled tube without collecting any of the organic or interphase layers. Use a P200 micropipette to prevent the solution from “jumping.”
7. Add an additional equal volume 24:1 chloroform:isoamyl alcohol to each tube and mix through shaking by hand for 30 s.
8. Centrifuge for 3 min at 18000 rcf.
9. Transfer the aqueous phase to a new labeled tube without collecting any of the organic or interphase layers. Leave a thin top layer above the interphase to minimize cross-phase contamination.
10. Add 60% (of the resulting volume of your sample) room temperature 100% isopropanol and mix gently by inverting three times.
11. Allow the samples to precipitate overnight at room temperature (>8 h).

Stopping Point (End of Day 1 or Day 2)

1. Centrifuge for 30 min at 18000 rcf. (Note: arrange the tubes identically so the pellet should be located in the tube at the same place.)
2. While centrifuging:
   1. Prepare 200 μL 70% ethanol per sample and place in a −20°C freezer.
   2. Set heat block to 56°C.
3. Carefully remove the supernatant with a micropipette without removing the pellet. (Note: the pellet may be hard to see.)
4. Add 200 μL cold (−20°C) 70% ethanol to each sample.
5. Gently invert each tube and ensure that the ethanol touches all the inner surfaces; keep an eye on the pellet (it can get caught under the cap).
   1. If the pellet dislodges, re-centrifuge for 5 min at 18000 rcf to recreate pellet.
6. Carefullly remove as much of the supernatant as possible with a micropipette without removing the pellet.
7. With the caps open, incubate the samples at 56°C for 5 min on the heat block under the fume hood (with fume hood on). The remaining ethanol should evaporate, and there should be no ethanol before beginning next step.
8. Add 50 μL TE (light) buffer or PCR-grade H_2_O per sample.
   1. Make sure the DNA pellet is in TE buffer by gently turning or moving it with the pipette tip.
9. Incubate the samples at 56°C for 5 min (this time with the caps closed) to suspend the pellets.
10. Store the samples at 4°C until they are ready to be used.

**1. 8x Bead Cleanup Protocol (for 50-μL samples)**

1. Thaw DNA sample to room temperature.
2. Add 90 μL of beads to the DNA sample.
3. Mix thoroughly by vortexing and/or pipetting up and down multiple times.
4. Incubate sample at room temperature for 5 min (DNA binds to beads).
5. Place sample on magnet rack to capture the beads (incubate at room temperature until the liquid is clear).
6. Remove and discard supernatant (do not touch the beads).
7. Keeping sample on magnet rack, add 50 μL of 80% ethanol.
8. Incubate the sample on the magnet at room temperature for ~1 min.
9. Carefully remove and discard the ethanol. Try to remove all residual ethanol without disturbing the beads.
10. Dry the beads at room temperature for 3–5 min (3 min 33 s is recommended) or until all the ethanol has evaporated.
11. Remove the sample from the magnet.
12. Resuspend the beads in 50 μL of elution buffer (PCR-grade water).
13. Incubate the sample for 2 min to elute the DNA off of the beads.
14. Place the sample on a magnet to capture the beads. Incubate until the liquid is clear.
15. Transfer the clear supernatant to a new tube.
16. DONE!

**KAPA HyperPlus Kit Library Construction Protocol, with modifications from the original bolded and in green (references to page numbers direct to the manufacturer instructions)**

Notes:

- First-time users should refer to Appendix 2: Optimization of Fragmentation Parameters (p. 16) before trying this kit, as standard fragmentation parameters may not result in the optimal size distribution for libraries prepared from your specific DNA samples. Precious samples should not be used when evaluating this kit. Instead, parameters should be optimized with a non-precious, bulk DNA sample that is representative of the actual samples to be processed.
- *If your DNA samples contain EDTA,* please consult Appendix 2: Handling of DNA Samples Containing EDTA (p. 16), as well as Important Parameters: Input DNA (p. 4) before starting this protocol.
- This protocol does not include size selection. Please refer to Appendix 1 (p. 15) for a detailed double-sided size selection protocol that may be included after ligation or after amplification.
- Always ensure that KAPA cleanup beads are fully equilibrated to room temperature and fully resuspended before use.
  1. **Enzymatic Fragmentation**

*If the DNA samples contain EDTA, perform a 3X bead-based cleanup with KAPA cleanup beads to remove EDTA prior to fragmentation.* Please refer to the relevant Technical Data Sheet (KR1705 or KR1245) for a detailed DNA cleanup protocol.

Alternatively, prepare a sufficient volume of appropriately diluted Conditioning Solution (5 μL per DNA sample, plus excess). Refer to Table 2 (p. 4) for guidelines on the dilution of the Conditioning Solution.

- 1. Dilute the amount of dsDNA to be used for library construction as follows:
     - If the DNA preparation does not contain EDTA, dilute in 10 mM Tris-HCl (pH 8.0–8.5) in a total of 35 µL.
     - If the DNA preparation does contain EDTA, dilute in the EDTA-containing buffer in which samples are currently suspended, in a total of 30 μL. To each reaction with 30 μL of EDTA-containing DNA, add 5 μL of diluted Conditioning Solution.
     - **1000/Qubit value = amount of DNA (μL) needed.**
  2. Mix by gentle vortexing or pipetting up and down.
  3. Assemble each fragmentation reaction on ice by adding the components in this order:

| Component | Volume | **½ Rctn** |
| --- | --- | --- |
| Double-stranded DNA (with Conditioning Solution, if needed) | 35 µL | **35 μL** |
| KAPA Frag Buffer (10X)* | 5 µL | **2.5 μL** |
| KAPA Frag Enzyme* | 10 µL | **5 μL** |
| Total volume: | 50 µL | **42.6 μL** |

*The KAPA Frag Buffer and Enzyme may be pre-mixed and kept on ice prior to reaction setup, and dispensed as a single solution. Please note the volume of buffer is less than the volume of enzyme in this reaction.

- 1. Vortex gently and spin down briefly. Return the plate/tube(s) to ice. Proceed immediately to the next step.
  2. Incubate in a thermocycler, pre-cooled to 4°C and programmed as outlined below. A heated lid is not required for this step. If used, set the temperature of the heated lid to ≤50°C.

| Step | Temp | Time |
| --- | --- | --- |
| Pre-cool block | 4°C | N/A |
| Fragmentation | 37°C | See table below |
| HOLD | 4°C | ∞ |

| Mode fragment length | Incubation time at 37°C* | Optimization range |
| --- | --- | --- |
| **1000 bp** | **3 min** | **Unknown** |
| 600 bp | 5 min | 3–10 min |
| 350 bp | 10 min | 5–20 min |
| 200 bp | 20 min | 10–25 min |
| 150 bp | 30 min | 20–40 min |

*These parameters are a good starting point for high-quality genomic DNA. Please refer to Appendix 2: Optimization of Fragmentation Parameters (p. 16) for guidelines on how to optimize fragmentation time and temperature. If incubation times longer than the recommended range are needed, samples likely contain inhibitors which impact the fragmentation efficiency. Bead-based DNA cleanup, prior to fragmentation, is recommended over longer fragmentation times.

- 1. Transfer reactions to ice, and proceed immediately to End Repair and A-tailing (step 2).
  2. **End Repair and A-tailing**

Two End Repair and A-Tailing Enzyme Mixes are provided for the End Repair and A-tailing step — see Important Parameters for further guidelines.

*IMPORTANT! The KAPA HyperPrep/Plus End Repair and A-tailing Buffer may contain white precipitates when thawed. Ensure the buffer is thoroughly vortexed until the precipitate has been resuspended. Heat at 37*°*C for 5–10 min, if indicated.*

*IMPORTANT! The KAPA HyperPlus End Repair and A-Tailing Enzyme Mix is NOT COMPATIBLE with mechanically fragmented (e.g., Covaris-sheared) input DNA used in KAPA HyperPrep workflows and usage thereof will result in library preparation failure.*

- 1. In the same plate/tube(s) in which enzymatic fragmentation was performed, assemble each End Repair and A-tailing reaction as follows:

| Component | Volume | **½ Rctn** |
| --- | --- | --- |
| Fragmented, double-stranded DNA | 50 µL | **42.6 µL** |
| End Repair & A-Tailing Buffer* | 7 µL | **3.5 µL** |
| HyperPrep/HyperPlus ERAT Enzyme Mix** | 3 µL | **1.5 µL** |
| Total volume: | 60 µL | **47.6 µL** |

*The buffer and enzyme mix should preferably be pre-mixed and added in a single pipetting step. Premixes are stable for ≤24 h at room temperature, for ≤3 days at 2°C to 8°C, and for ≤4 weeks at −15°C to −25°C.

**Use either the HyperPrep ERAT Enzyme Mix (existing chemisty) or the HyperPlus ERAT Enzyme Mix (enhanced chemistry).


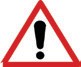


- 1. Vortex gently and spin down briefly. Return the reaction plate/tube(s) to ice. Proceed immediately to the next step.
  2. Incubate in a thermocycler programmed as outlined below. A heated lid is required for this step. If possible, set the temperature of the heated lid to ~85°C (instead of the usual 105°C).

| Step | Temp | Time |
| --- | --- | --- |
| End repair and A-tailing | 65°C* | 30 min |
| HOLD | 4°C** | ∞ |

*Both the fragmentation and end repair enzymes are inactivated at 65°C. When reactions are set up according to recommendations, additional fragmentation should be negligible. The brief period of end repair is sufficient for enzymatically fragmented DNA.

**If proceeding to the adapter ligation reaction setup without any delay, the reaction may be cooled to 20°C instead of 4°C.

- 1. Proceed immediately to Adapter Ligation (step 3).
  2. **Adapter Ligation**

*IMPORTANT! The KAPA HyperPrep Ligation Buffer contains a high concentration of PEG 6000 and is very viscous. Small PEG 6000 droplets may be visible when thawed and require special attention during pipetting. Ensure the buffer is thoroughly vortexed until the droplets*  *have been resuspended. Heat at 37*°*C for 5–10 min, if indicated.*

- 1. Dilute adapter stocks to the appropriate concentration, as outlined in Table 4 (p. 5).
  2. In the same plate/tube(s) in which end repair and A-tailing was performed, assemble each adapter ligation reaction as follows:

| Component | Volume | **½ Rctn** |
| --- | --- | --- |
| End repair and A-tailing reaction product | 60 µL | **47.55 µL** |
| Adapter stock  (diluted as per Table 4 on p. 5) | 5 µL | **5 µL**** |
| PCR-grade water* | 5 µL | **2.5 µL** |
| Ligation Buffer* | 30 µL | **15 µL** |
| DNA Ligase* | 10 µL | **5 µL** |
| Total volume: | 110 µL | **72.5 µL** |

*The water, buffer and ligase enzyme should preferably be premixed and added in a single pipetting step. Premixes are stable for ≤24 h at room temperature, for ≤3 days at 2°C to 8°C, and for ≤4 weeks at −15°C to −25°C.

**Add Adapter Stock to each well.

**Note: Pull out beads and PEG/NaCl 30 min prior to use (only for problematic samples).**

- 1. Mix thoroughly and centrifuge briefly.
  2. Incubate at **4°C** for **12 h.**

Note: To achieve higher conversion rates and library yields, particularly for low-input samples, consider increasing the ligation time to a maximum of 4 h at 20°C or overnight at 2°C to 8°C. Please note that longer ligation times may lead to increased levels of adapter-dimer. Adapter concentrations may have to be optimized if ligation times are extended significantly.

- 1. Proceed immediately to Post-ligation Cleanup (step 4).
  2. **Post-ligation Cleanup**
  3. In the same plate/tube(s), perform a 0.8X bead- based cleanup by combining the following:

| Component | Volume | **½ Rctn** |
| --- | --- | --- |
| Adapter ligation reaction product | 110 µL | **72.5 µL** |
| KAPA cleanup beads | 88 µL | **44 µL** |
| Total volume: | 198 µL | **116.5 µL** |

- 1. Mix thoroughly by vortexing and/or pipetting up and down multiple times.
  2. Incubate the plate/tube(s) at room temperature for **10 min** to bind DNA to the beads.
  3. Place the plate/tube(s) on a magnet to capture the beads. Incubate until the liquid is clear.
  4. Carefully remove and discard the supernatant.
  5. Keeping the plate/tube(s) on the magnet, add **100 µL of 80% ethanol**.
  6. Incubate the plate/tube(s) on the magnet at room temperature for **1 min**.
  7. Carefully remove and discard the ethanol.
  8. Keeping the plate/tube(s) on the magnet, add **100 µL of 80% ethanol.**
  9. Incubate the plate/tube(s) on the magnet at room temperature for **1 min**.
  10. Carefully remove and discard the ethanol. Try to remove all residual ethanol without disturbing the beads.
  11. Dry the beads at room temperature for 3–5 min, or until all of the ethanol has evaporated. *Caution: over-drying the beads may result in reduced yield.*
  12. Remove the plate/tube(s) from the magnet.

**PEG/NaCl clean-up (only for problematic samples):**

- **Re-suspend beads in 50 μL 10 mM Tris (25 μL for ½ reaction)**
- **Wait 2 min**
- **Add 40 μL PEG/NaCl (20 μL for ½ reaction)**
- **Wait 10 min**
- **Repeat ethanol washes (step 4.4)**
  1. Resuspend the beads:
     - In **22 µL of elution buffer** (10 mM Tris-HCl, pH 8.0–8.5) to proceed with Library Amplification (step 5), or
     - In 55 µL of elution buffer (10 mM Tris-HCl, pH 8.0–8.5) to proceed with double-sided size selection (Appendix 1).
  2. Incubate the plate/tube(s) at room temperature for 2 min to elute DNA off the beads.
  3. Place the plate/tube(s) on a magnet to capture the beads. Incubate until the liquid is clear.
  4. Transfer the clear supernatant to a new plate/tube(s):
     - To proceed with Library Amplification (step 5), transfer 20 µL of supernatant, or
     - To proceed with double-sided size selection (Appendix 1), transfer 50 µL of supernatant.
  5. **Library Amplification**

Note: Please refer to Important Parameters: Library Amplification (p. 7) and the KAPA NGS Library Preparation Technical Guide (available on request from Technical Support at sequencing.roche.com/support) for more information on optimizing library amplification.

- 1. Assemble each library amplification reaction as follows:

| **Component** | **Volume** |
| --- | --- |
| **KAPA HiFi HotStart ReadyMix (2X)** | **25 µL** |
| **H_2_O** | **3 µL** |
| **i5 and i7 primers** | **1 µL each** |
| **Total volume:** | **29 µL** |

**Or add reagents individually**

| **Component** | **Volume** |
| --- | --- |
| **5x HiFi w/Mg** | **10 µL** |
| **dNTPs** | **1.25 µL** |
| **H_2_O** | **15.75 µL** |
| **Hotstart Polymerase** | **1 µL** |
| **i5 and i7 primers** | **1 µL each** |
| **Total volume:** | **29 µL** |

- 1. Mix thoroughly and centrifuge briefly.
     Amplify using the following cycling protocol:

| Step | Temp | Duration | Cycles |
| --- | --- | --- | --- |
| Initial denaturation | 98°C | 45 s | 1 |
| Denaturation | 98°C | 15 s | Minimum number required for optimal  amplification  (Table 5 or 6) |
| Annealing* | 60°C | 30 s |  |
| Extension | 72°C | 30 s |  |
| Final extension | 72°C | 1 min | 1 |
| HOLD | 4°C | ∞ | 1 |

*Optimization of the annealing temperature may be required for non- standard (i.e., other than Illumina TruSeq) adapter/primer combinations.

- 1. Proceed directly to Post-amplification Cleanup (step 6).
  2. **Post-amplification Cleanup**
  3. In the library amplification plate/tube(s), perform a 1X bead-based cleanup by combining the following:

| Component | Volume |
| --- | --- |
| Library amplification reaction product | 50 µL |
| KAPA cleanup beads | 50 µL |
| Total volume: | 100 µL |

- 1. Mix thoroughly by vortexing and/or pipetting up and down multiple times.
  2. Incubate the plate/tube(s) at room temperature for 5–15 min to bind DNA to the beads.
  3. Place the plate/tube(s) on a magnet to capture the beads. Incubate until the liquid is clear **(10 min)**.
  4. Carefully remove and discard the supernatant.
  5. Keeping the plate/tube(s) on the magnet, add 200 µL of 80% ethanol.
  6. Incubate the plate/tube(s) on the magnet at room temperature for **1 min**.
  7. Carefully remove and discard the ethanol.
  8. Keeping the plate/tube(s) on the magnet, add 200 µL of 80% ethanol.
  9. Incubate the plate/tube(s) on the magnet at room temperature for **1 min**.
  10. Carefully remove and discard the ethanol. Try to remove all residual ethanol without disturbing the beads.
  11. Dry the beads at room temperature for 3–5 min, or until all of the ethanol has evaporated. *Caution: over-drying the beads may result in reduced yield.*
  12. Remove the plate/tube(s) from the magnet.

**PEG/NaCl clean-up (only do for problematic samples):**

- **Re-suspend beads in 50 μL 10 mM Tris (25 μL for ½ reaction)**
- **Wait 2 min**
- **Add 40 μL PEG/NaCl (20 μL for ½ reaction)**
- **Wait 10 min**
- **Repeat ethanol washes (step 6.6)**
- **Place on magnet**
- **Remove 85 μL**
  1. Thoroughly resuspend the beads in an appropriate volume of elution buffer **(22 μL)** (10 mM Tris-HCl, pH 8.0–8.5)*.* Note: If proceeding with a second post-amplification cleanup, or double-sided size selection (Appendix 1), resuspend the beads in 55 µL of elution buffer.
  2. Incubate the plate/tube(s) at room temperature for 2 min to elute DNA off the beads.
  3. Place the plate/tube(s) on a magnet to capture the beads. Incubate until the liquid is clear.
  4. Transfer the clear supernatant **(20 μL)** to a new plate/ tube(s) and proceed with size selection (refer to Appendix 1), library QC, target capture or sequencing, as appropriate. Store purified, amplified libraries at 2°C to 8°C for 1–2 weeks, or at −15°C to −25°C.
