# Supplementary material for: Toward an open‐source 3D‐printable laboratory
Source: Appl Plant Sci. 2024 Jan 18;12(1):e11562. doi: 10.1002/aps3.11562 (PMC10873812; doi:10.1002/aps3.11562)
Supplement: Supplementary file 3 — Appendix S3. Step‐by‐step guide to assembly of all COBLE equipment listed in Table 1. [file APS3-12-e11562-s001.docx]

**Appendix S3.** Step-by-step guide to assembly of all COBLE equipment listed in Table 1.


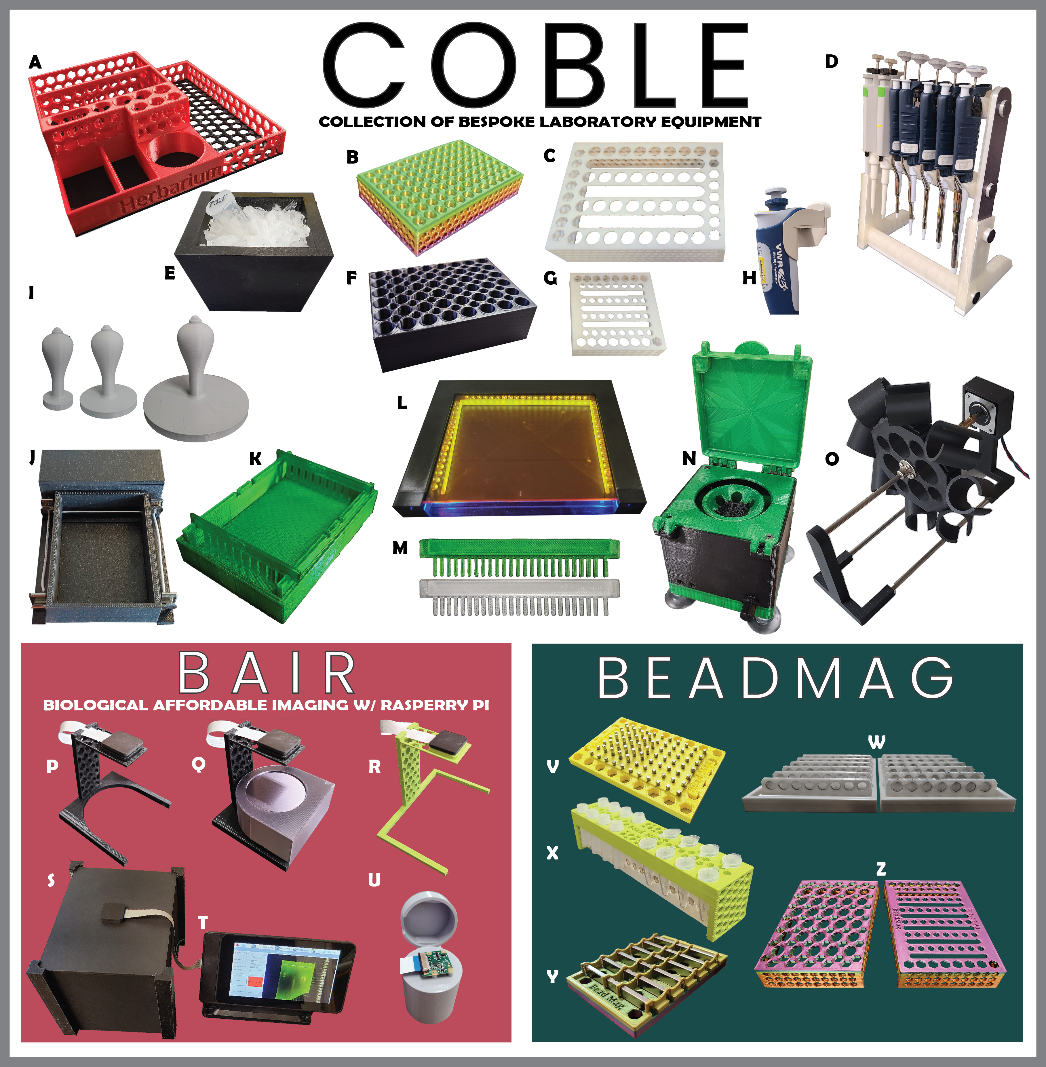


- [BAIR Imaging System (Raspberry Pi assembly & Camera Adapters)](#_BAIR_System:_Imaging)
- [Transilluminator](#_BAIR_System:_Transilluminator)
- [Gel Electrophoresis Unit](#_BAIR_System:_Gel)
- [BeadMag Separators](#_BAIR_System:_BeadMag)
- [Tabletop Mini Centrifuge](#_BAIR_System:_DIY)
- Sir Tumbalot Tube Tumbler
- [Additional 3D Printing Resources](#_Additional_3D_Printing)

# BAIR System

Bill of Materials

- 3D-printed parts (print whatever parts required for your specific needs; if you need something specific not created yet reach out to us!)
  - BAIR Camera Adapters
    - Transilluminator Hood
    - 96-well plate
    - 100-mm round plate
      - 60-mm Round Plate Holder
    - Microscope Adapters (Various Sizes)
  - Other designs we suggest
    - [Raspberry Pi Housing + Display Case](https://www.thingiverse.com/thing:1585924)
    - [Articulating Arm](https://www.prusaprinters.org/prints/3407-articulating-raspberry-pi-camera-mount-for-prusa-m)
      - BAIR Camera Housing
      - [NoIR Camera Housing](https://www.prusaprinters.org/prints/64679-raspberry-pi-camera-mount-for-ir-camera)
      - [Manual Focus Adjustment Camera Housing](https://www.prusaprinters.org/prints/64384-housing-for-the-raspberry-pi-standard-camera-modul) (tolerance needs improvement for the focus gear)
      - [G Clamp with Ball-socket connector](https://www.thingiverse.com/thing:4940731) (print in PETG or stronger)
- 1, Raspberry Pi 3B+ or 4 and 7” Screen (requires additional parts to function)
  - [Kit that includes all necessary pieces and display](https://www.canakit.com/canakit-raspberry-pi-desktop-kit-black.html)
    OR
  - [Power supply](https://www.amazon.com/CanaKit-Raspberry-Supply-Adapter-Listed/dp/B00MARDJZ4/ref=sr_1_1?crid=OGVDM6FEUU15&keywords=raspberry+pi+3b%2B+power+supply&qid=1646254220&sprefix=raspberry+pi+3b%2B+power+supply%2Caps%2C71&sr=8-1)
  - [MicroSD card](https://www.amazon.com/Raspberry-Pi-16GB-Preloaded-Noobs/dp/B01H5ZNOYG/ref=sr_1_3?crid=NT4D0NQHRA00&keywords=raspberry+pi+3b%2B+micro+sd+card&qid=1646254241&sprefix=raspberry+pi+3b%2B+microsd+card%2Caps%2C62&sr=8-3) (with NOOBS installed preferred; max 64GB capacity)
  - Wired [Mouse and Keyboard](https://www.amazon.com/Raspberry-Official-Keyboard-PepperTech-Digital/dp/B07RDGFRW4/ref=sr_1_2?crid=1WJKAJLHVCAQT&keywords=raspberry+pi+mouse&qid=1646254317&sprefix=raspberry+pi+mouse%2Caps%2C83&sr=8-2) (any brand works)
  - [Heat sinks for Raspberry Pi computer](https://www.amazon.com/JTRJ-Radiator-Cooling-Raspberry-Aluminum/dp/B09T5ZZ7P4/ref=sr_1_2?crid=HTFRX2BRRJ9N&keywords=raspberry+pi+heatsinks&qid=1646254379&sprefix=raspberry+pi+heatsinks%2Caps%2C70&sr=8-2) (copper or aluminum preferred)
  - [On/Off switch](https://www.amazon.com/LoveRPi-MicroUSB-Switch-Raspberry-Female/dp/B018BFWLRU/ref=sr_1_2?crid=3JL2HUUHDE7KE&keywords=raspberry+pi+power+switch&qid=1646254420&sprefix=raspberry+pi+power+switch%2Caps%2C85&sr=8-2) (optional)
- 1, [1m Ribbon Cable](https://www.amazon.com/A1-FFCs-Black-Raspberry-Camera/dp/B07J57LQQS/ref=sr_1_5?crid=2QNIOVMPYHJ3A&keywords=pi+camera+ribbon+cable&qid=1643673509&s=electronics&sprefix=pi+camera+ribbon+cabl%2Celectronics%2C89&sr=1-5)
- 1, Raspberry Pi compatible camera
  - [Raspberry Pi Camera V2](https://www.canakit.com/raspberry-pi-camera-v2-8mp.html?cid=usd&src=raspberrypi) (NoIR version available)
  - Arducam Pi Camera [V2 (8mp)](https://www.arducam.com/product-category/cameras-for-raspberrypi/raspberry-pi-camera-raspistill-raspvivid/raspberry-pi-camera-v2-imx219-8mp/) or [V3 (16mp)](https://www.arducam.com/16mp-autofocus-camera-for-raspberry-pi/) (NoIR versions also available)

Instructions

**Setting Up Your MicroSD Card**

- If you bought a microSD card with NOOBS pre-installed, skip this and move on to **“Assembling the Raspberry Pi”**, otherwise continue below
- Install the SD Card formatting software from
  - <https://www.sdcard.org/downloads/formatter_4/index.html>
- Plug your MicroSD card into your computer by inserting it into an SD card adapter (included with the purchase of most microSD cards) and inserting that into your SD card drive
- If your computer does not have an SD card drive, use an external card reader
- Using the SD Card Formatting Software, format your microSD card
- Download the New Out Of Box Software (NOOBS) directly from Raspberry Pi; this can take upwards of an hour.
  - <https://www.raspberrypi.org/downloads/noobs/>
- Once the download has finished, unzip the folder and move all of the files from the unzipped folder directly to your microSD card.
- Safely eject the microSD card from your computer.
- Your microSD card is ready for use!

**Assembling the Raspberry Pi**

-
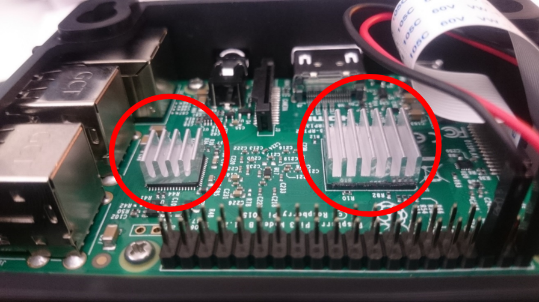
Begin by attaching the heat sinks to the motherboard.

- They attach using adhesive that should already be on the bottom of each heat sink.
-
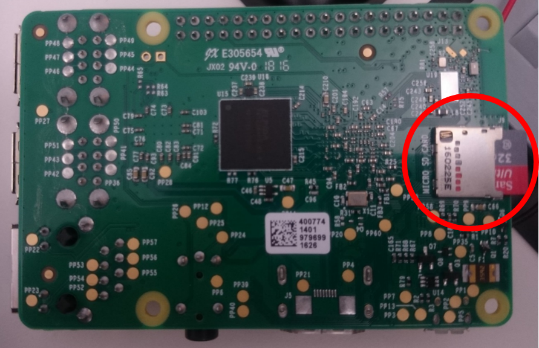
Insert the microSD card loaded with NOOBS into the slot on the back of the motherboard.
-
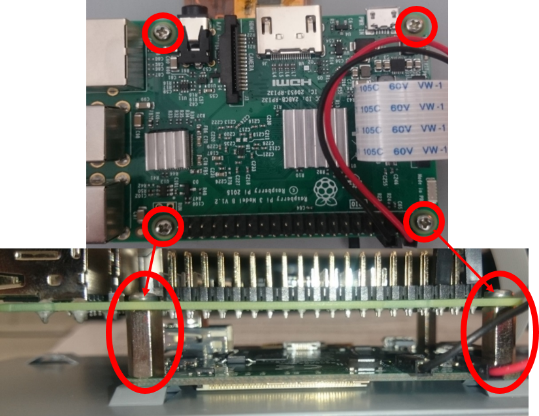
Attach the motherboard to the screen using the pegs and screws provided.
- Be sure that wording on both the motherboard and screen board are facing the same direction.
-
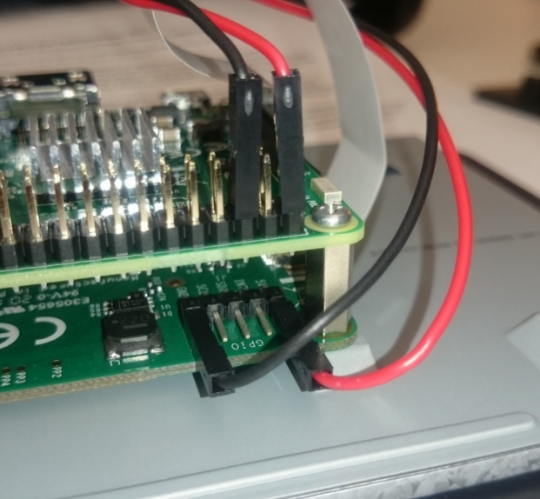
Attach the power cables to the motherboard and screen exactly as pictured.
- The black cable attaches to the 3rd post from the end on the motherboard and the far left on the screen board.
- The red cable attaches to the corner post of the motherboard and the far right of the screen board.
-
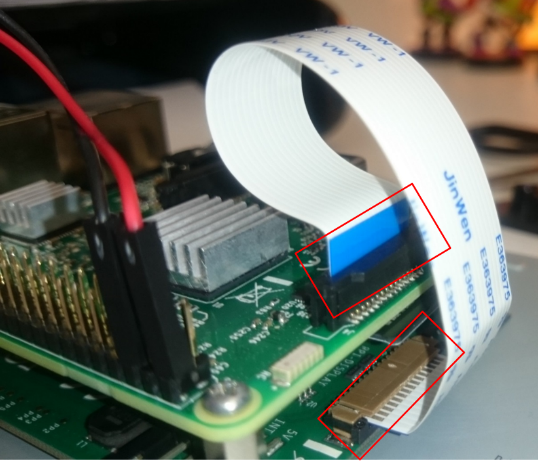
Attach the small ribbon cable to the motherboard and screen board exactly as pictured.
- Put the case onto the assembled Raspberry Pi screen/motherboard.
- Be sure to line up the holes of the case with the holes for the screws.
- Be sure that none of the cables are being crushed between the case and the Raspberry Pi screen/motherboard.
-
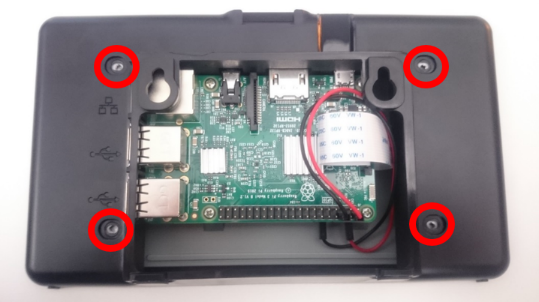
Start all four screws, but do not tighten them.
- Once all four screws have been started, tighten until they are snug.
-
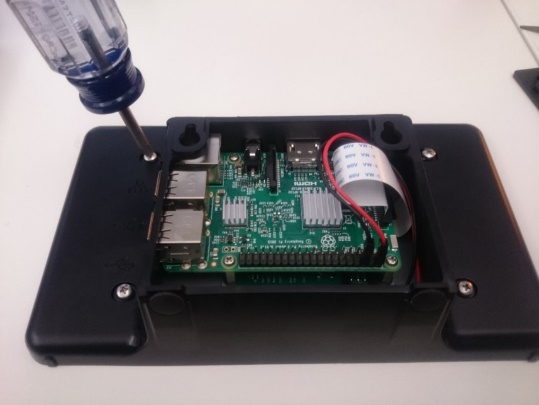
Be careful not to overtighten them or you could damage the screen.
-
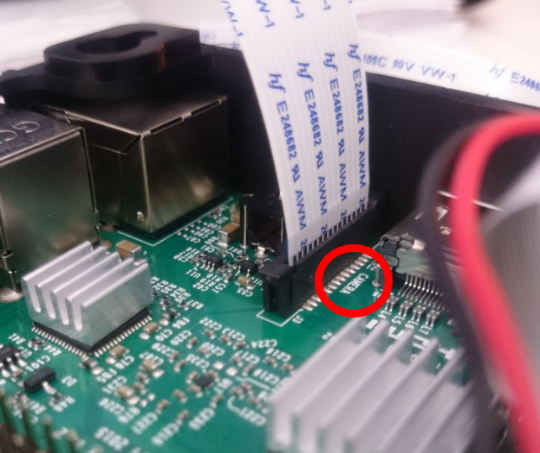
Insert the long ribbon cable for the camera into the camera slot on the motherboard.
- Be sure the contacts are facing the word camera on the motherboard, or the camera will not work.
- Do not force it in; it is a tight fit. Wiggle the cable back and forth gently while applying pressure until it slips into place. Try not to crease the ribbon cable.
-
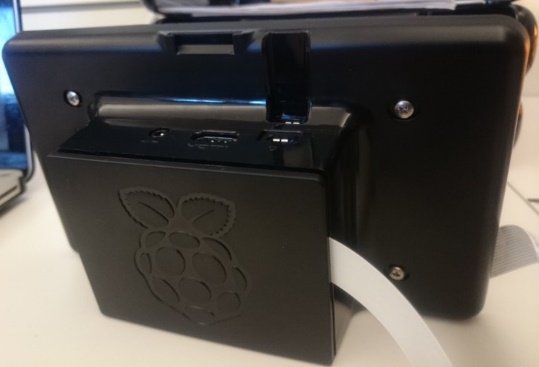
Run the ribbon cable out the side of the case and put the back cover on (it snaps into place).
- Be sure not to crush any cables.
- The Pi Camera is inherently focused at infinity rather than close up.
  - This means that things that are far away from the camera will be in focus rather than things close to the camera.
- How to manually focus the Pi Camera.
  - What you’ll need:
    - White Camera focusing tool (comes with camera) OR
    - 2 small, fine-tipped needle nose pliers
    - Cushiony surface such as a mouse pad
    - Aluminum foil to cover the mouse pad (reduces static)
- Hold the lens with one pair of pliers and grip the darker black circular part of the lens with the other pair of pliers or the camera focusing tool.
- Hold the pliers firmly so they don’t slip and scratch the board or lens.
- Turn the camera lens counterclockwise 90 degrees to get it to its closest focal point.
- This video does a good job of explaining this process: <https://www.youtube.com/watch?v=u6VhRVH3Z6Y>
-
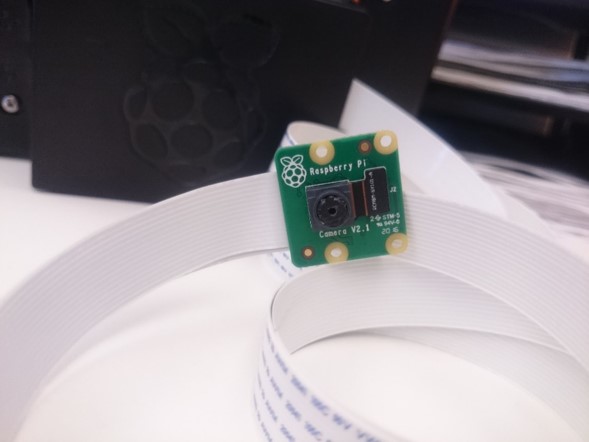
Insert the ribbon cable into the Pi Camera.
- Be sure the contacts on the ribbon cable are facing the camera lens or the camera will not work.
- Again, be gentle and wiggle the cable back and forth until it is in place.
- Congratulations! You have successfully assembled your Raspberry Pi Imaging System.
- Now it is time to turn it on and complete the installation of the operating system and all the software you will need.

**Installing the OS**

- The Operating System (OS) you will be installing is called Raspbian.
  - There are many other options provided, so be sure you choose the correct one.
- Before you turn on your Raspberry Pi for the first time, make sure you are in a place with a good WiFi connection.
  - If you do not have WiFi, you can plug an ethernet cable directly into the Raspberry Pi.
- You may need a wired mouse and keyboard for the installation process.
  - If your Bluetooth Keyboard/Mouse does not have a USB dongle, you will need wired versions.
- Plug the power cable into the micro USB plug next to the HDMI port.
  -
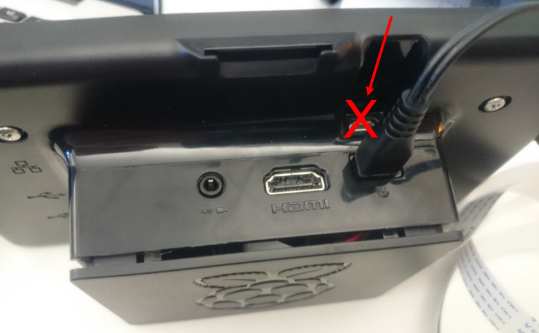
Ignore the second micro USB plug here, plugging the power cable here will only power the screen, not the computer.
- **Important Note: The screen will likely display upside down, this is easily remedied after the OS is installed.**
- The next screen that loads should have a list of Operating Systems. Alternatively, you may be prompted to update your version of NOOBS.
  - Connect to your WiFi and download the latest version by following the prompts on screen.
  - After the latest versions download, you should be on a screen with a list of Operating Systems.
- Select “Raspbian” and click “Install,” then click “Yes” to begin the installation process.
- During the installation process, there is an option at the bottom of the screen to change the language of your computer and the configuration of your keyboard. If necessary, go ahead and change it.
- Once the OS has installed successfully, click “OK” and the Raspberry Pi should restart on its own.

**Configuring Your OS**

- After the Raspberry Pi has restarted, you will come to a screen titled “Raspberry Pi Software Configuration Tool (raspi-config).”
  - Use the arrow keys on your keyboard to select Option 4 “Internationalization Options,” then press Enter.
  - Change your Locale to match your location.
    - Select the locale that works best for all of those that will be using the Raspberry Pi.
    - You can select multiple locales if necessary.
    - Use the space key to select and deselect locales.
    - Press Enter when finished to confirm your selections.
    - Press Enter again to finish changing your locale.
- You should now be back at the “Raspberry Pi Software Configuration Tool (raspi-config)” screen.
  - Use the arrow keys on your keyboard to select Option 4 “Internationalization Options,” then press Enter.
    - Change your time zone to match your location.
      - Select the geographic location that is closest to you and press Enter.
      - Select your time zone from the list and press Enter.
- Repeat the above steps to adjust keyboard layout if necessary.
- Select Option 5, “Enable Camera,” then press Enter.
  - Select “Enable” and press Enter.
- You should be back at the main screen again; select “Finish” and reboot the Raspberry Pi.
- **Rotating Your Screen**
-
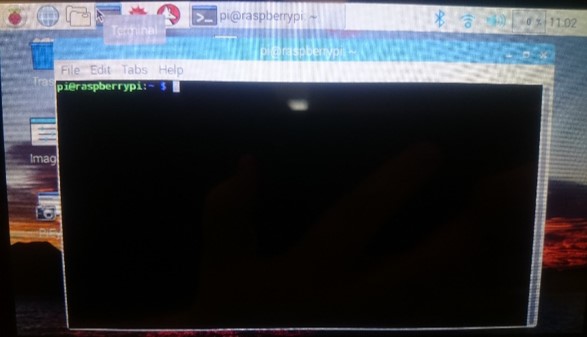

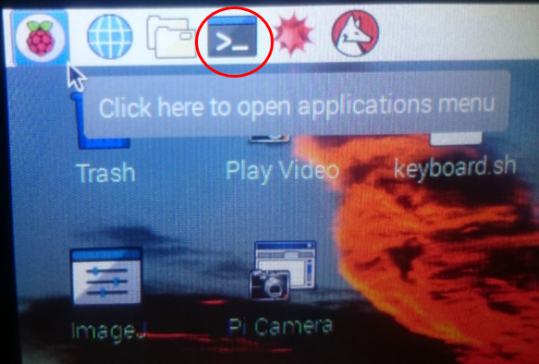
Open the Command Line (circled in red).
- Type cd /boot
- Type sudo nano config.txt
- Add the following to a new line in the document lcd_rotate=2
- Hit Control+x, then y, then Enter to save your changes.
- Restart for changes to take effect.

**Installing and Running the Imaging Software**

- Open the Command Line.
- Type cd /home/pi/your/destination/folder/
- To clone (i.e. download) the BAIR git repository type git clone https://github.com/PeterPieGH/BAIR.git
- Alternatively, download the Python script manually from <https://github.com/PeterPieGH/BAIR.git> into your destination folder.
- Type cd BAIR to change into the folder with the Python script.
- Type python3 bair_app.py to start the GUI.
- Set your interval time by typing in a number and then pressing Enter on your keyboard (time between taking an image).
- Set your total time by typing in a number and then pressing Enter on your keyboard (total amount of time you want the time lapse to run).
- If desired, set your ISO and shutter speed manually.
- If desired, set your preferred image resolution (the maximum setting will vary depending on the type of camera you use).
- Set the directory you want the images to be saved in. (Note: Do not try to save to an external hard drive or flash drive directly from the GUI.)
- Set your preferred filename prefix.
- Click Show Preview to ensure your camera is focused on the subject.
- Click Take Snapshot to take a single still image.
- If video is checked, BAIR will automatically create a time-lapse video using the images it takes during the total time; the longer the total time, the longer it will take to create the video. (Note: Do not close the BAIR GUI while the video is processing or it will corrupt the video file.)
-
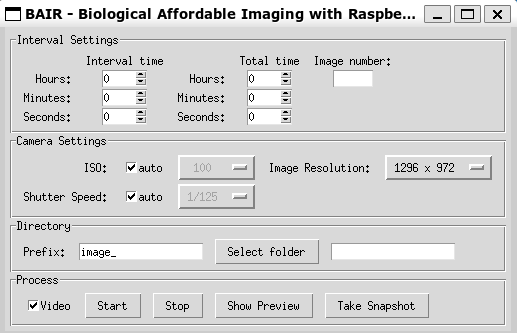
Click Start to begin your time lapse!

# Transilluminator

Bill of Materials

- 3D-printed parts (<https://www.printables.com/model/45750-coble-collection-of-bespoke-laboratory-equipment>)
  - LED Holder
  - Electronics Cover
  - Diffuser
  - Left and Right Endcaps
- 36, [5-mm through-hole 471-nm LEDs](https://www.mouser.com/ProductDetail/828-OVLGB0C6B9)
- [2422 Amber Acrylic (filter material)](https://www.amazon.com/gp/product/B07D9S36XH/ref%3Dppx_yo_dt_b_asin_title_o00_s00?ie=UTF8&psc=1)
- 36, [90 Ohm Resistors (preferred)](https://www.mouser.com/ProductDetail/Vishay-BC-Components/SFR25H0009099FR500?qs=%2Fha2pyFaduiDg1AR%2Fqhorvld3TvSdFJ1bhPt%2Fr0rpVLHytcl2AXKr14TX2YHvxrU) or 100 Ohm ½ watt resistors
- [22-gauge solid hookup wire](https://www.amazon.com/ELECTRONIX-EXPRESS-Solid-Hook-Wire/dp/B00DRGAQ0I/ref=sr_1_5?crid=10SSJ67V9JW8A&keywords=22+gauge+hookup+wire&qid=1646245526&s=hi&sprefix=22+gauge+hookup+wire%2Ctools%2C68&sr=1-5)
- 1, [6-foot/2-meter USB cable with one Type A end](https://www.amazon.com/Monoprice-Male-Micro-28AWG-Cable/dp/B001UWN81A/ref=sr_1_1?keywords=6ft+usb+A+cable&qid=1646245649&refinements=p_85%3A2470955011&rnid=2470954011&rps=1&sr=8-1)
- 1, [5-V, 1-amp USB Wall Adapter](https://www.amazon.com/Charger-Adapter-VectorTech-2-Pack-Samsung/dp/B07GMVPCX5/ref=sr_1_3?crid=3GYY8GS7ZM5PL&keywords=5v+1amp+usb+power+supply&qid=1646250781&refinements=p_85%3A2470955011&rnid=2470954011&rps=1&sprefix=5v+1amp+usb+power+supply%2Caps%2C61&sr=8-3)
- Solder
- Solder flux
- Soldering Iron
- Wire Stripper/Cutter

Instructions

- Insert LEDs into 3D-printed part to ensure fit, then remove.
-
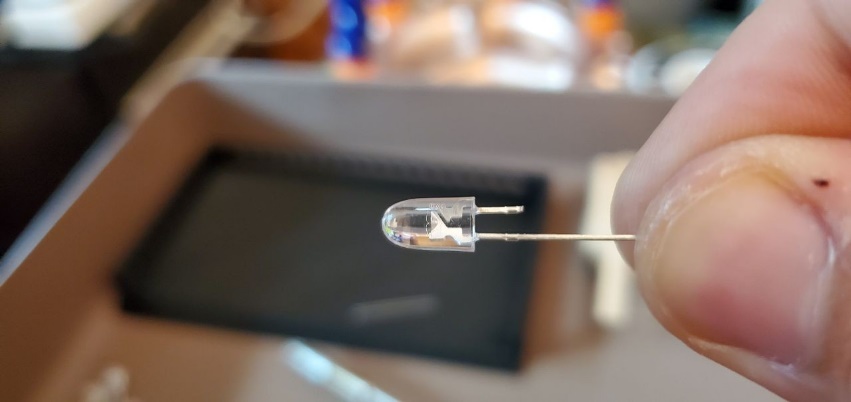
Clip the positive leg of all the LEDs just above the small flat mark about 5 mm down the leg from the bulb base.
- Clip the resistors to the same length.
-
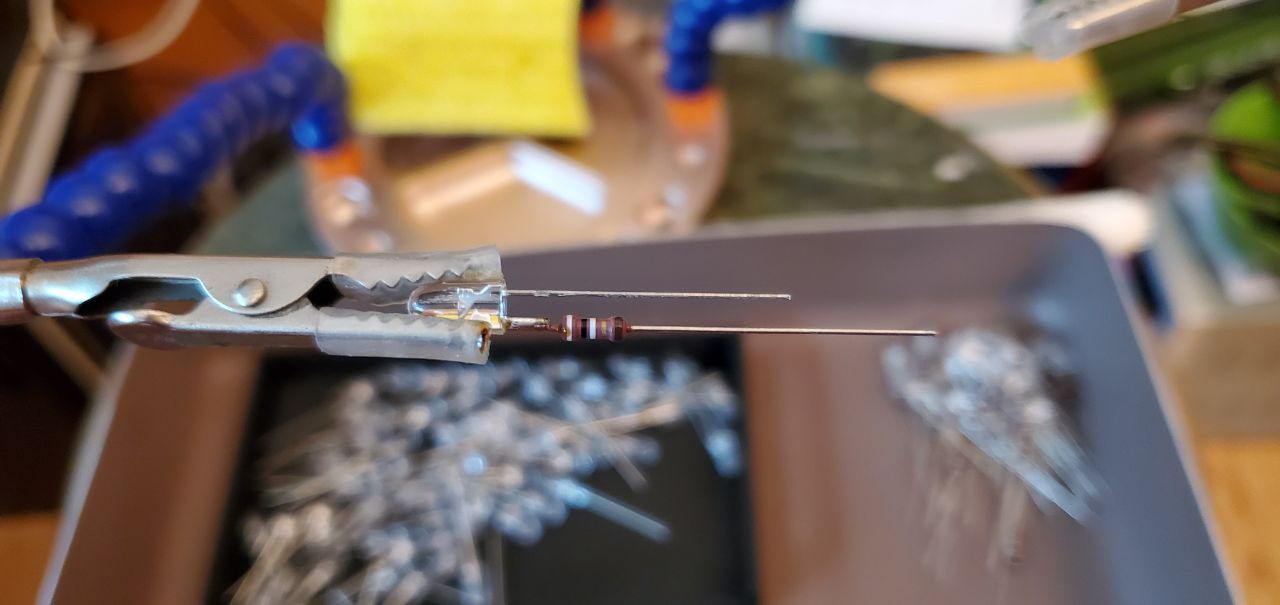
Solder a resistor to the positive leg of each LED.
-
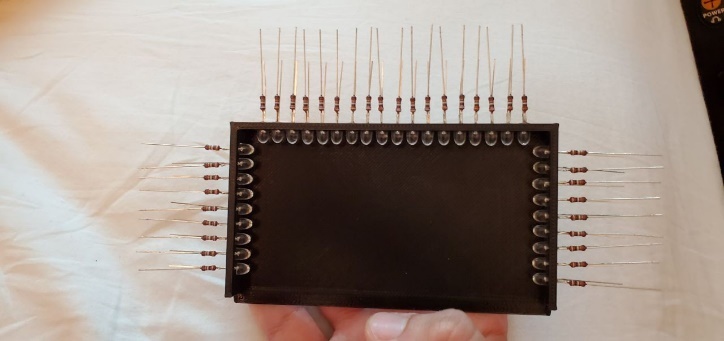
Orient all the LEDs resistor side up along the holes of the LED Holder
-
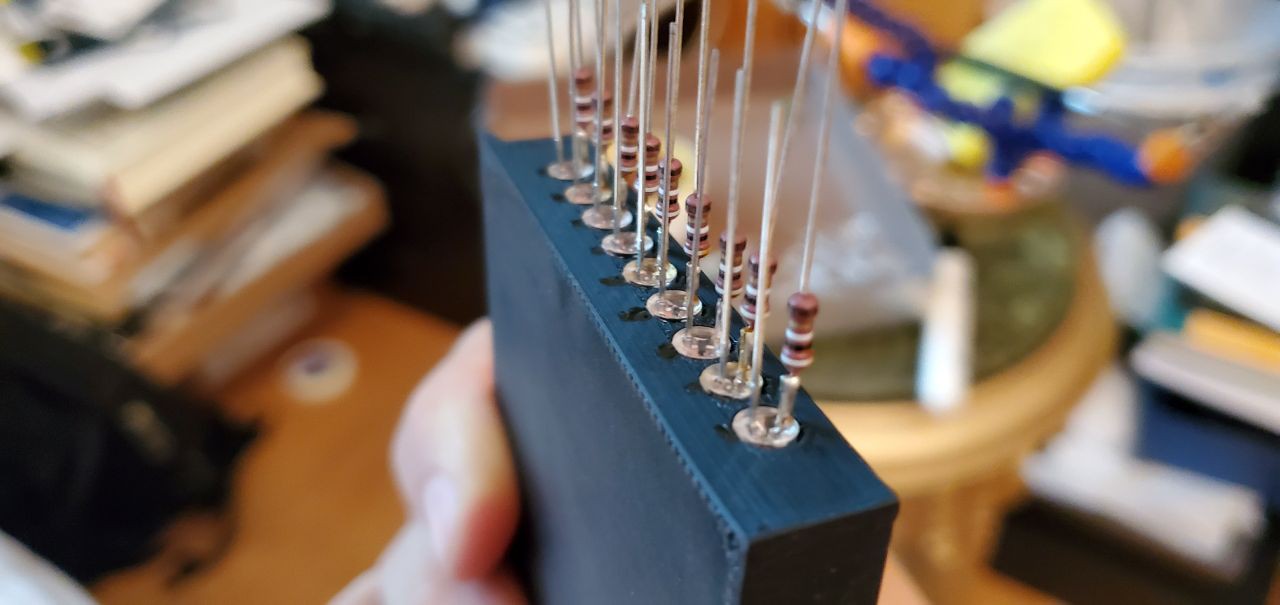
For non-skirted LEDs, add a small drop of cyanoacrylate (super) glue to keep the LEDs in place.
- Strip two 12” 22-AWG gauge wires and twist them into a solid strand.
- Solder the wire such that all the positive legs are soldered in a parallel formation. (Applying ﬂux just below the resistor end opposite the LED bulb works best.)
-
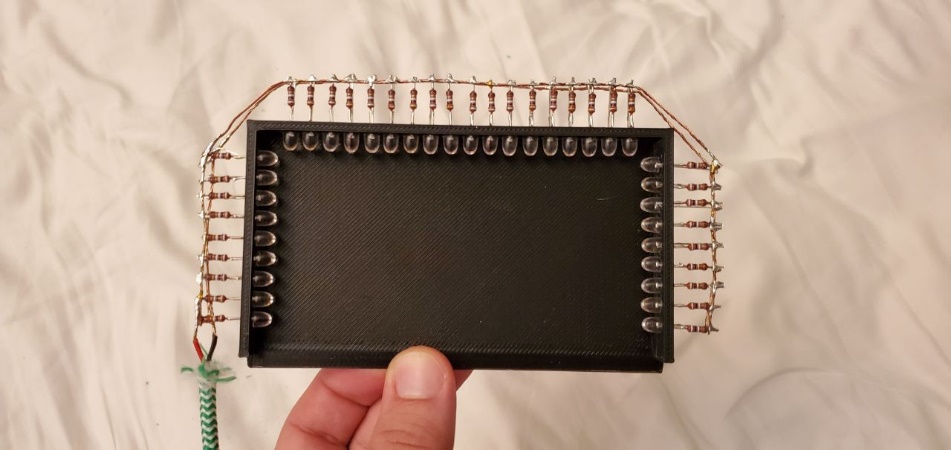
Ensure each of the LED legs are connected in parallel to the other corresponding legs of similar polarity: one rail of positive legs, one rail of negative, so that all LED units are now in parallel. Final trimmed version shown here:
- Strip the 6-ft USB cable at the end opposite the male Type A connector.
- Isolate the red and black wires.
- Cut the green and white data wires cleanly to avoid shorting.
- Strip and tin ~5 mm of copper from each of the red and black wires.
-
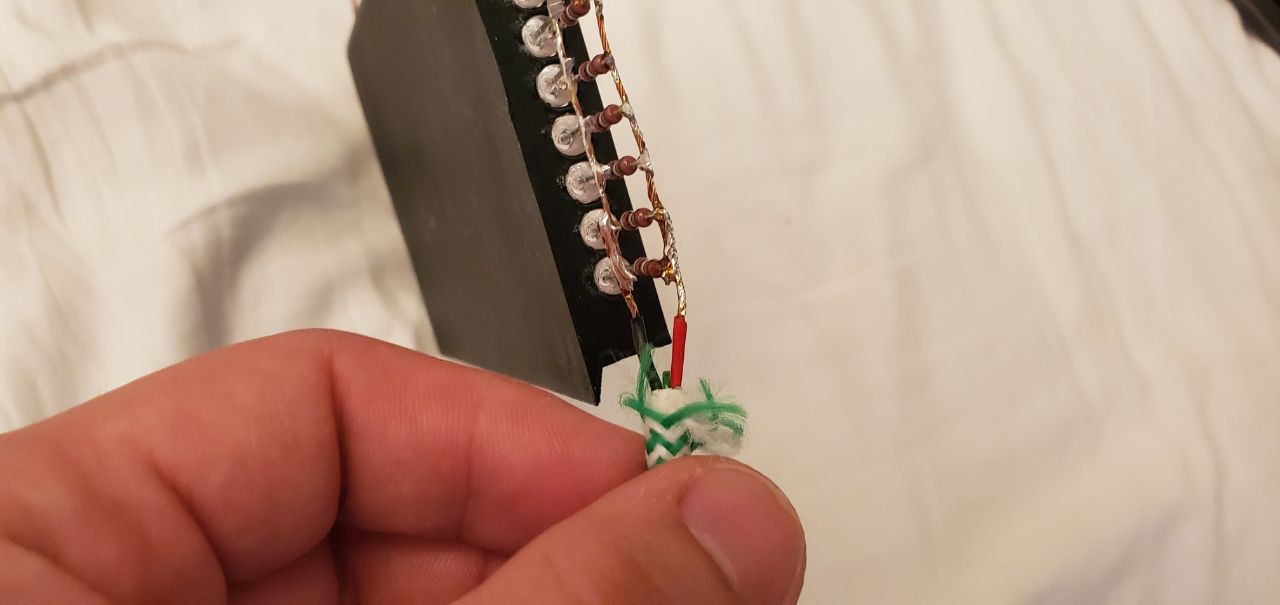
Solder them to the rails of the LEDs with the red attaching to the top rail with all of the resistors and the black wire attaching to the rail without resistors on them, as shown in this image:

- Score and cut the acrylic sheet to 140–145 mm long and 115–120 mm wide.
- Insert the USB A cable through the slot of the Electronics Cover.
- Slide the LED Holder into the Electronics Cover, being careful not to pinch the USB cable.
- Slide the acrylic sheet into place between the Electronics Cover and LED Holder.
- Glue the Left and Right Endcaps to the Electronics Cover, then slide the 2422 Acrylic Filter out to ensure you do not glue it in place.
-
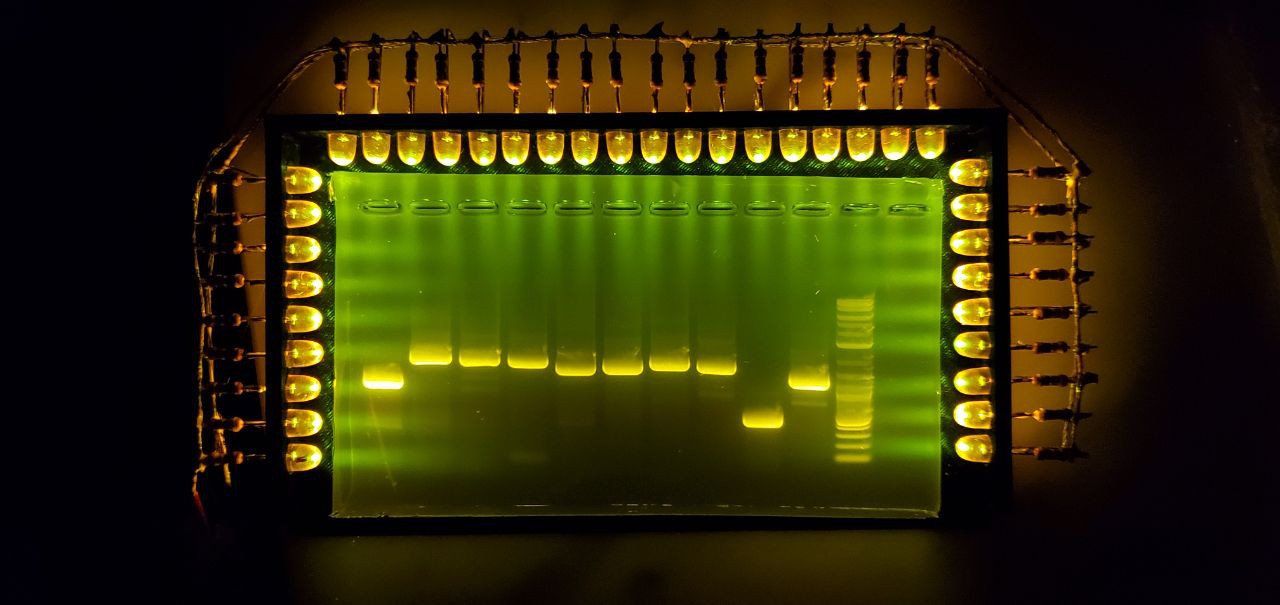
Once all glue has dried, replace the Acrylic Filter and plug the USB cable to the 5-V, 1-amp wall adapter to power on the transilluminator.

# Gel Electrophoresis Unit

*This design is in the very early stages of alpha testing and needs significant development and improvement.

Bill of Materials

- 3D-printed parts
  - Gel Boat Holder SC
  - Gel Boat SC
  - Gel Comb 6 wells SC
  - Electronics Housing SC
  - Electronics Housing Lid SC
  - Electrophoresis Gel Box SC
  - Gel Boat Holder MM
  - Gel Boat MM
  - Combs MM (10, 12, 15, and 16 wells)
- 1, [Booster Converter Module](https://www.amazon.com/dp/B07XG323G8/ref=cm_sw_r_apanp_YeBPJ1VYFcyhE)
- [2-mm stainless steel rods](https://www.amazon.com/gp/product/B00QBYPT6Y/ref=ppx_yo_dt_b_search_asin_title?ie=UTF8&psc=1)
- 4, [Battery Clips](https://www.amazon.com/dp/B07LGG8BCV/ref=cm_sw_r_apanp_P8mRyeU3EVfpy?th=1)
- [22-gauge solid hookup wire](https://www.amazon.com/ELECTRONIX-EXPRESS-Solid-Hook-Wire/dp/B00DRGAQ0I/ref=sr_1_5?crid=10SSJ67V9JW8A&keywords=22+gauge+hookup+wire&qid=1646245526&s=hi&sprefix=22+gauge+hookup+wire%2Ctools%2C68&sr=1-5)
- 1, [6-foot/2-meter USB cable with one Type A end](https://www.amazon.com/Monoprice-Male-Micro-28AWG-Cable/dp/B001UWN81A/ref=sr_1_1?keywords=6ft+usb+A+cable&qid=1646245649&refinements=p_85%3A2470955011&rnid=2470954011&rps=1&sr=8-1)
- 1, [5-V, 1-amp USB Wall Adapter](https://www.amazon.com/Charger-Adapter-VectorTech-2-Pack-Samsung/dp/B07GMVPCX5/ref=sr_1_3?crid=3GYY8GS7ZM5PL&keywords=5v+1amp+usb+power+supply&qid=1646250781&refinements=p_85%3A2470955011&rnid=2470954011&rps=1&sprefix=5v+1amp+usb+power+supply%2Caps%2C61&sr=8-3)
- [Black Silicone Sealant](https://www.amazon.com/gp/product/B000AL6WLA/ref=ppx_yo_dt_b_search_asin_title?ie=UTF8&th=1)
- Solder
- Solder flux
- Soldering Iron

Instructions

- Cut two pieces of 2-mm stainless steel bars as wide as the electrophoresis tank. Use an emery board or nail file to smooth the cut as to not scratch user or snag gloves.
- Route the cut rods through the two holes in the electrophoresis tank.
- Center the rods with the tank and seal the rods in place with a small smear of silicone sealant. Be sure to carefully fill all the way around each side of the tank wall to ensure there are no leaks. Remove any excess sealant from the rod on either side to ensure good electrical contact with the tabs soldered next.
- Strip and tin the ends of four 6”-long 22-gauge solid-core wires.
- Solder a wire onto each of the four battery clips on the tab opposite the textured surface.
- Press each of the soldered battery clips firmly onto each of the vertical walls that protrude out from the arms of the electrophoresis tank. Orient the clips such that they form a “V” channel with the textured clip facing away from the base of the electrophoresis tank. These will be the landing troughs the electrodes will sit on.
- Route the wires such that they feed into the openings in the base. Keep track of which wires go to which clips.
- Twist and solder together the tinned leads of the two closest clip wires, these will be the negative electrode. Solder these to the “OUTPUT(-)” side of the booster module.
- Twist and solder together the tinned leads of the two furthest electrode clips, these will be the positive electrode. Solder these to the “OUTPUT(+)” side of the booster module.
- Cut a USB cable such that only a USB Type A male end remains while the other side of the cable is cut off entirely.
- Strip back the shielding of the cut end of the USB cable to reveal four wires. The color order is as follows: Black (GND), Red (5 V), Green and White (Data in/out). Cut back the green and white wires but leave 2” of the red and black wires as we will be using them in the next step.
- Strip and tin the red 5-V and black ground wire.
- Snake the USB cable’s cut end through the hole in the back of the electrophoresis tank.
- Solder the red 5-V USB wire to the “INPUT(+)” side of the booster module.
- Solder the black ground USB wire to the “INPUT(-)” side of the booster module.
- Use electrical tape to insulate any exposed wiring, tuck in the booster module carefully so as not to break the connections, and firmly place the lid onto the system.
- Test by filling the tank with 25 mL of buffer, plug in the USB to any standard USB receptacle, and place multimeter leads as close to each of the electrodes as possible without making contact. There should be a voltage above 10 V; this will vary depending on the buffer being used. For best results, aim for the highest voltage across the two electrodes. This may require adjusting the buffer concentration as there is ample room for experimentation.
-
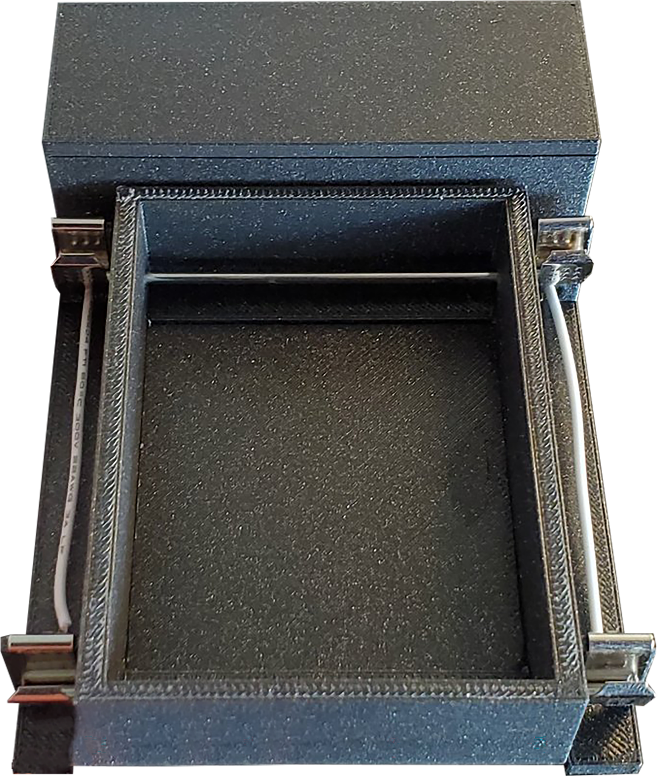
To shut off the device, simply unplug from USB wall adapter.
- For the latest build info, parts list, and STL files, please visit <https://github.com/Binomica-Labs/Hardware> and look for the MiniGel system, as well as other hardware projects of interest.

# BeadMag Separators

Bill of Materials

- 3D-printed parts
  - 1.5/2-mL Separator
    - 1.5/2-mL Tube Rack
    - 1.5/2-mL Magnet Block (6, 60 x 10 x 3-mm rectangular magnet)
    - 1.5/2-mL Magnet Block (10, 40 x 10 x 5-mm rectangular magnet)
  - Skirted Plate Separator
    - 48-Well (0.5-mL) Tube Rack
    - 30-Well (1.5-mL) Tube Rack
    - 96-Well Magnet Block (48, 8 x 1-mm round magnet)
    - 96-Well Magnet Block (48, 9 x 2-mm round magnet)
    - 96-Well Magnet Block (24, 20 x 10 x 5-mm rectangular magnet)
    - 96-Well Magnet Block (12, 40 x 10 x 5-mm rectangular magnet)
  - Microtitre Magnet Block
- Magnets required to make all versions:
  - 48, 5–10-mm long x 3-mm diameter cylindrical
  - 6, 60 x 10 x 3-mm rectangular
  - 22, 40 x 10 x 5-mm rectangular
  - 24, 20 x 10 x 5-mm rectangular
  - 48, 8 x 1-mm or 9 x 2-mm round

Instructions

- Purchase N52 strength magnets for faster bead separation.
- Slide the magnets into the appropriate slot on the 3D-printed Magnet Block.
- Add a drop of super glue to keep magnets in place if they pop out (required for microtiter block).


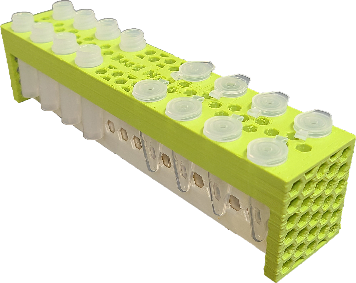

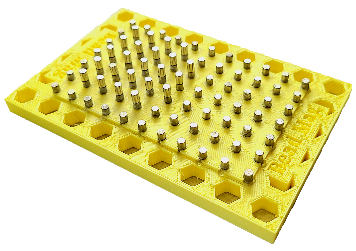

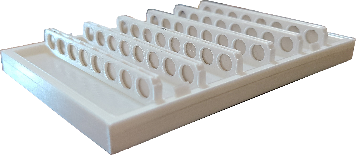

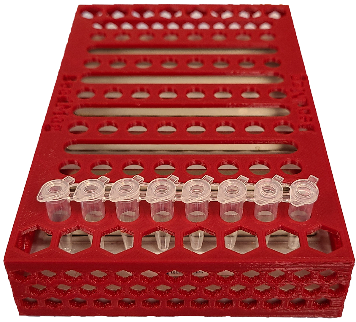

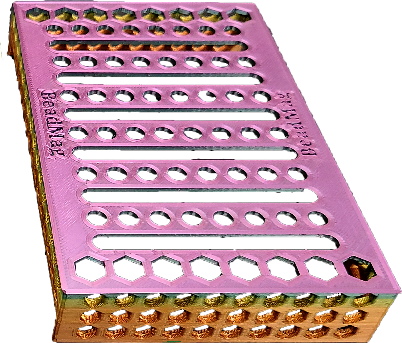

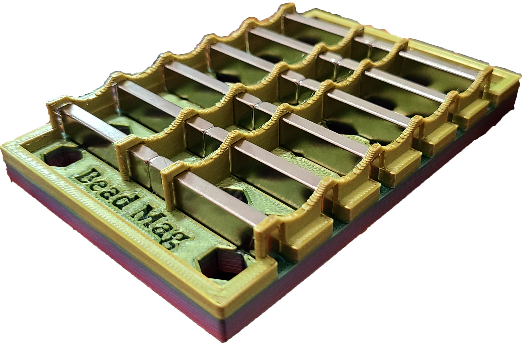

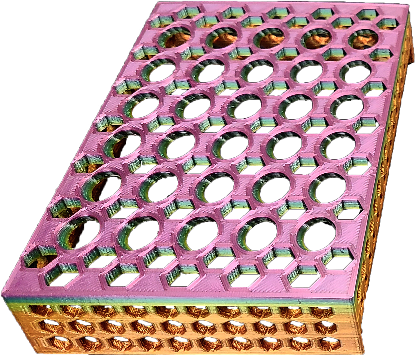


# DIY Tabletop Centrifuge

Bill of Materials

- 3D-printed parts
  - Centrifuge Lid
  - Centrifuge Containment Lid
  - Large Motor and Small Motor versions available
    - Centrifuge Body
    - Centrifuge Spill Case
    - Centrifuge Tube Holder
    - Centrifuge Motor Housing
    - Centrifuge Containment Lid Legs
  - VWR C-1200 Centrifuge Motor Housing
- 1, 6–12-V mini DC motor high torque [(large motor)](https://www.amazon.com/gp/product/B01M58POHF/ref=ppx_yo_dt_b_asin_title_o05_s00?ie=UTF8&psc=1) [(small motor)](https://www.amazon.com/gp/product/B07CKT5ZQM/ref=ppx_yo_dt_b_asin_title_o04_s00?ie=UTF8&psc=1)
- 1, [T85 SPST Rocker Switch](https://www.amazon.com/gp/product/B07S2QJKTX/ref=ppx_yo_dt_b_asin_title_o07_s00?ie=UTF8&psc=1)
- 1, [3–35-V DC Speed Controller 5A 90W](https://www.amazon.com/gp/product/B07GP72BWV/ref=ppx_yo_dt_b_asin_title_o08_s01?ie=UTF8&psc=1)
- 6, [Molex wire terminals](https://www.amazon.com/MOLEX-19164-0017-TERMINAL-FEMALE-pieces/dp/B011OB2P3S?ref_=Oct_d_obs_d_306729011&pd_rd_w=7FwfF&pf_rd_p=8000bc8c-c3b0-4816-9f00-5038ff54385c&pf_rd_r=H7C7C49HS33C80S9G8TQ&pd_rd_r=2286a723-09db-416d-b31a-e754dc034d7e&pd_rd_wg=MFb9c&pd_rd_i=B011OB2P3S)
- 1, [12-V DC power supply 2A 24W adapter](https://www.amazon.com/gp/product/B01HCRUGPW/ref=ppx_yo_dt_b_asin_title_o00_s01?ie=UTF8&psc=1)
- 4, [M6 Suction Cup feet](https://www.amazon.com/gp/product/B07CZVMRF2/ref=ppx_yo_dt_b_asin_title_o09_s00?ie=UTF8&psc=1)
- 1, [AC 250V 5A SPDT 3 pin microswitch](https://www.amazon.com/gp/product/B00MFRMFS6/ref=ppx_yo_dt_b_asin_title_o00_s01?ie=UTF8&psc=1)
- [22-gauge solid hookup wire](https://www.amazon.com/ELECTRONIX-EXPRESS-Solid-Hook-Wire/dp/B00DRGAQ0I/ref=sr_1_5?crid=10SSJ67V9JW8A&keywords=22+gauge+hookup+wire&qid=1646245526&s=hi&sprefix=22+gauge+hookup+wire%2Ctools%2C68&sr=1-5)
- Solder
- Solder flux
- Soldering Iron

Instructions

- Cut six, 14 cm lengths of 22-gauge solid hookup wire.
- Strip and tin ~5 mm of each end of all the wire lengths.
-
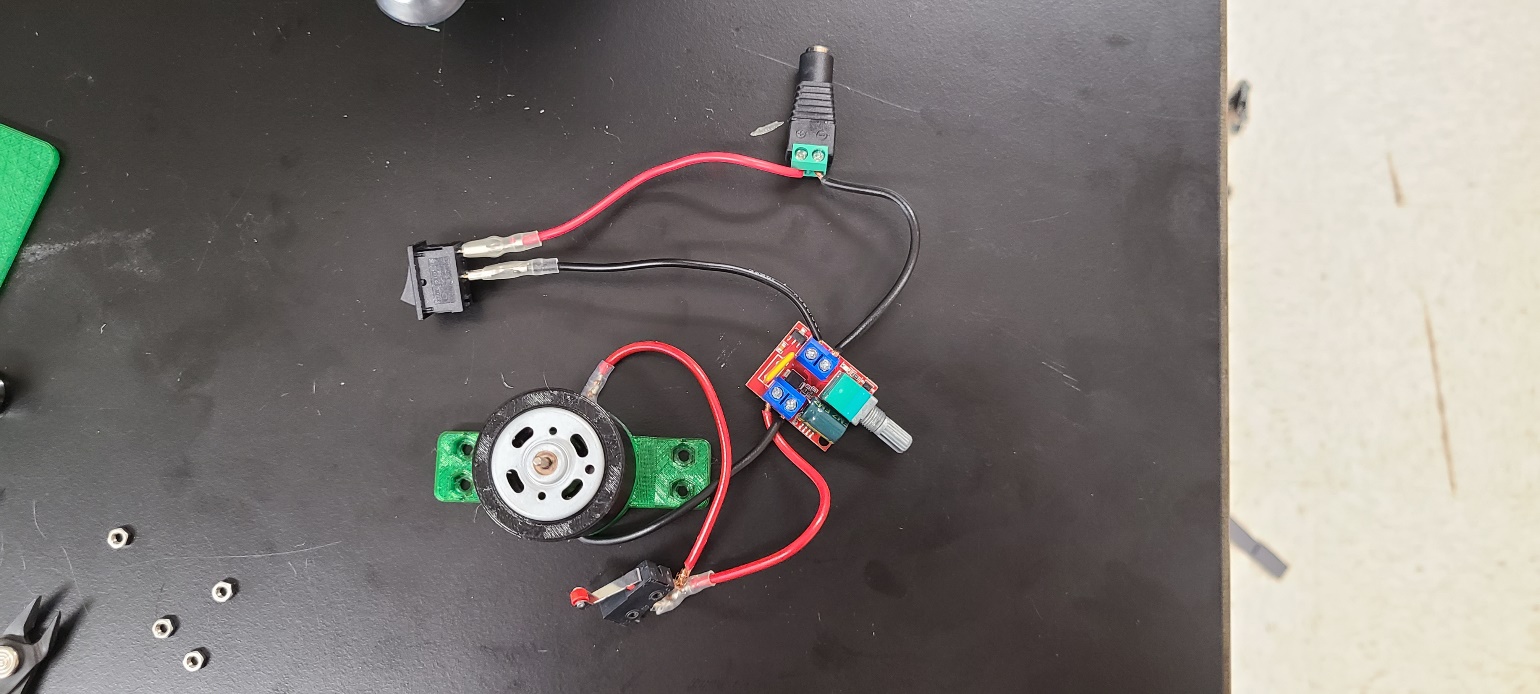
Solder Molex wire terminals to both ends of one length of wire and to only one end of four lengths of wire.
- Attach your wires as shown in the image.
  - Connect the positive terminal of the motor to the positive “C” terminal of the micro switch with the length of wire with two Molex terminals soldered to it.
  - Connect a wire with a single Molex terminal to the negative terminal of the motor, insert the other end on the negative terminal of the motor connector on the speed controller; tighten the screw on the speed to controller to lock the wire in place.
  - Connect a wire with a single Molex terminal to the “NO” terminal on the microswitch and insert the other end to the positive terminal of the motor connector on the speed controller; tighten the screw on the speed to controller to lock the wire in place.
  - Using the wire with no Molex terminals, connect the negative terminal of the power supply adapter to the negative terminal on the DC In connector on the speed controller; tighten the screws on the speed to controller and power adapter to lock the wire in place.
  - Connect one of the remaining two wires to the positive DC In terminal on the speed controller (use the end without a Molex terminal) ; tighten the screw on the speed to controller to lock the wire in place.
  - Connect the final wire to the positive terminal on the power adapter; tighten the screw on the power adapter to lock the wire in place.
-
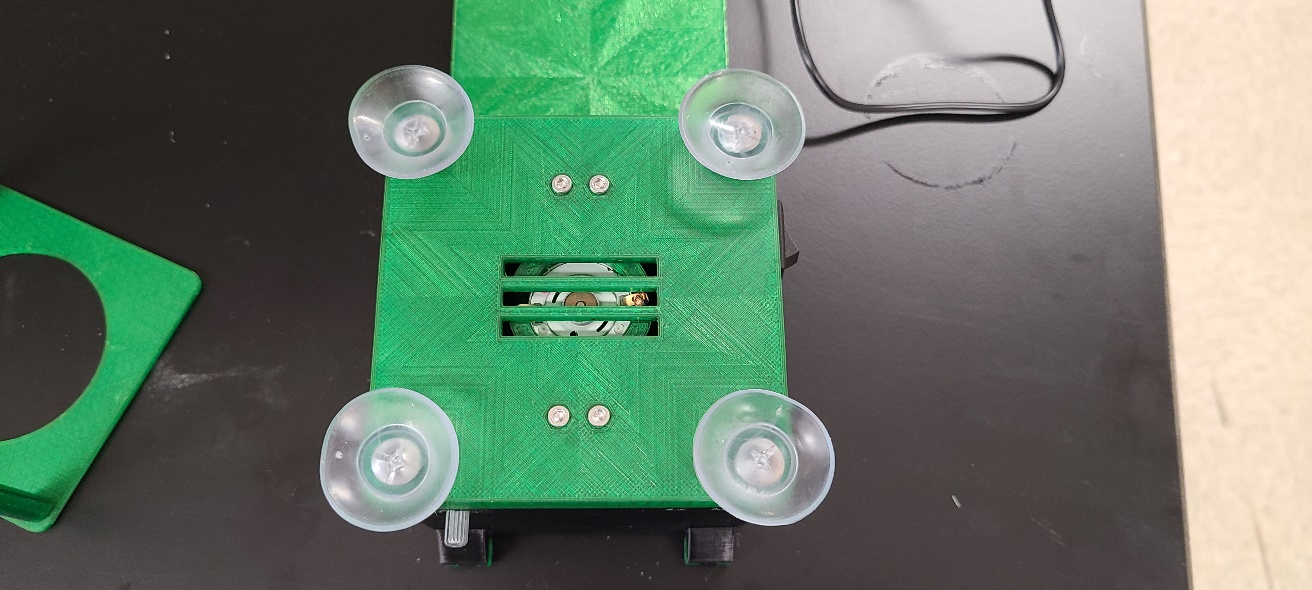
Screw the M6 Suction Cup feet into the corner holes on the bottom of the Centrifuge Body.
- Slide the motor into the 3D-printed Motor Housing piece through the bottom until the motor body (silver part) is flush with the top of the Motor Housing. (It should be a very snug fit; if it’s too tight, sand down the interior of the Motor Housing a little bit at a time until it slides in.)
- Insert four M3 hex nuts into the slots of the Motor Housing.
- Place the Motor Housing into the Centrifuge Body, being sure not to crush or disconnect any of the wiring.
- Without flipping the Centrifuge Body upside down (hex nuts will fall out), insert and tighten four M3 bolts (~8 mm length) through the bottom of the Centrifuge Body.
- Gently insert the Speed Controller through the slot at the front right of the Centrifuge Body.
- Gently insert the micro switch into the slot at the front left of the Centrifuge Body so the tallest part of the plunger is in the corner.
- Line up the holes in the front left of the Centrifuge Body and screw two 10 mm length M3 countersunk bolts through the micro switch. (Don’t force this as you can damage the micro switch.)
- Run the positive motor wire and the positive speed controller wire through the hole at the back left part of the Centrifuge Body, clip them onto the rocker switch.
- Insert the microswitch into the Centrifuge Body until it clicks into place.
-
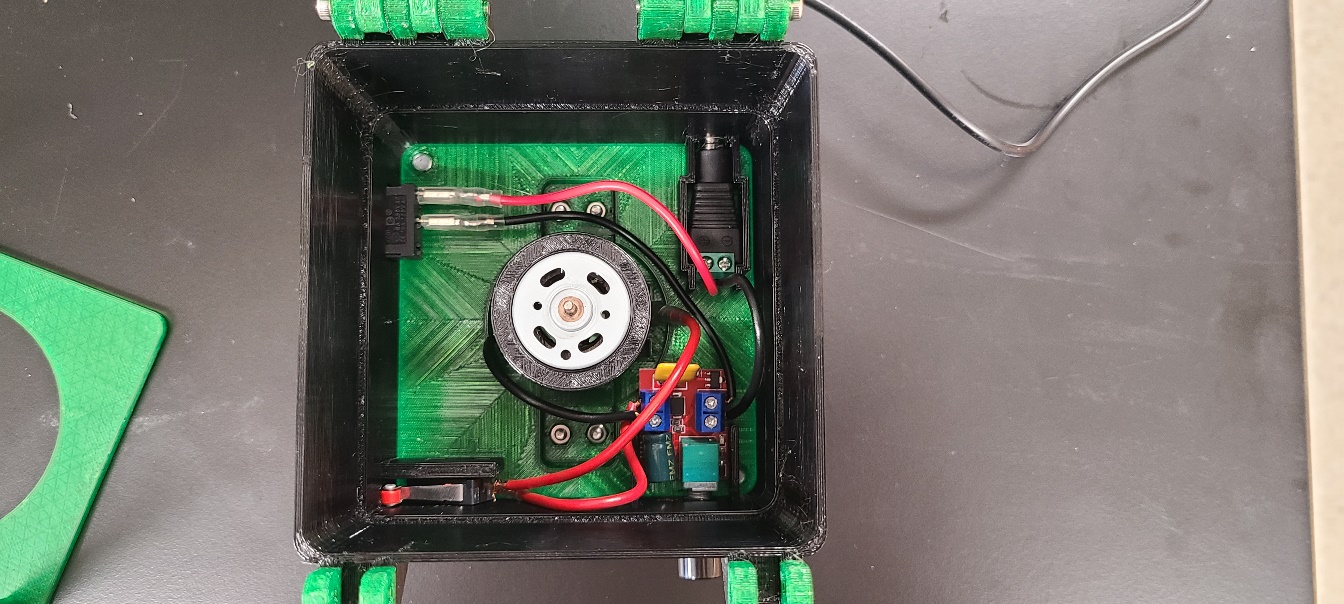
Gently insert the power adapter into the back right bracket, being sure it slots into the hole on the back of the Centrifuge Body.
- Test to make sure there are no shorts.
  - Plug the power cable into an outlet then insert the plug into the power adapter.
  - Turn the rocker switch into the On position – if a small LED on the speed controller does not turn on there is a short or something is disconnected.
  - Turn the speed controller nob clockwise until it clicks on.
  - Push down on the plunger of the micro switch to confirm the motor turns on.
  -
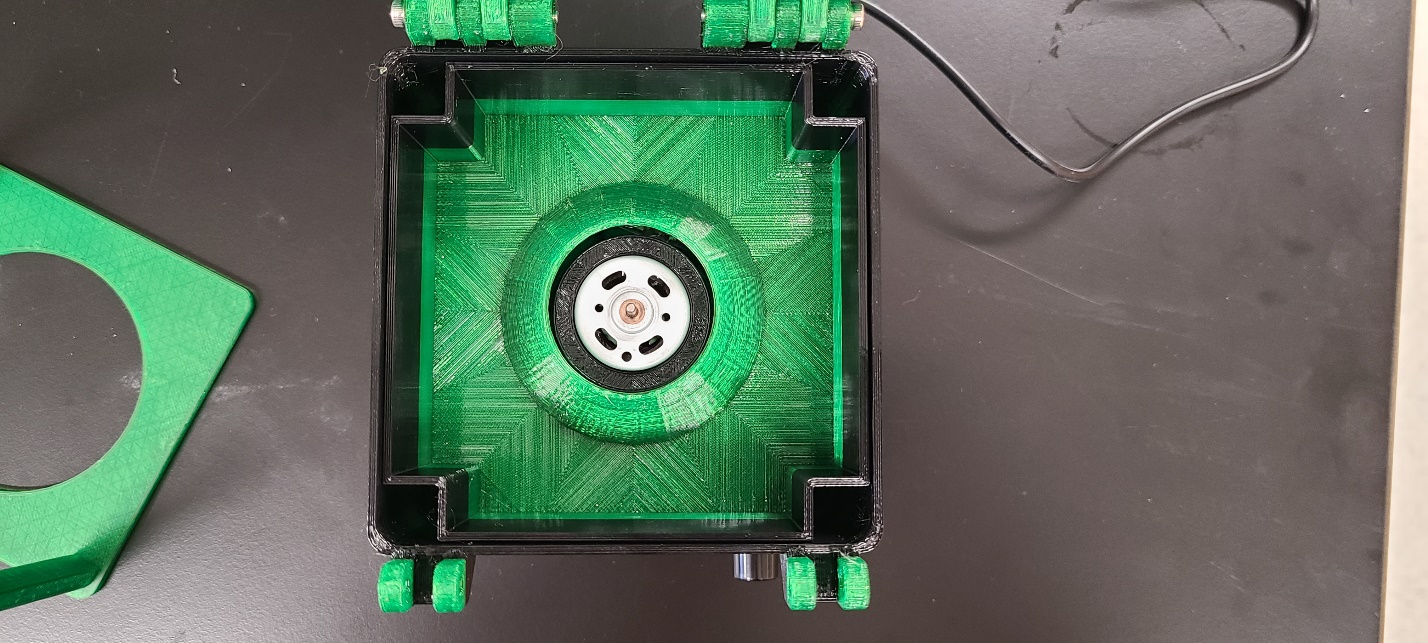
If everything works, proceed to the next step; if something does not work, use a multimeter to determine where the short/disconnection is.
- Insert the Centrifuge Spill Case into the Centrifuge Body. (It should act like a bowl in case a tube breaks.)


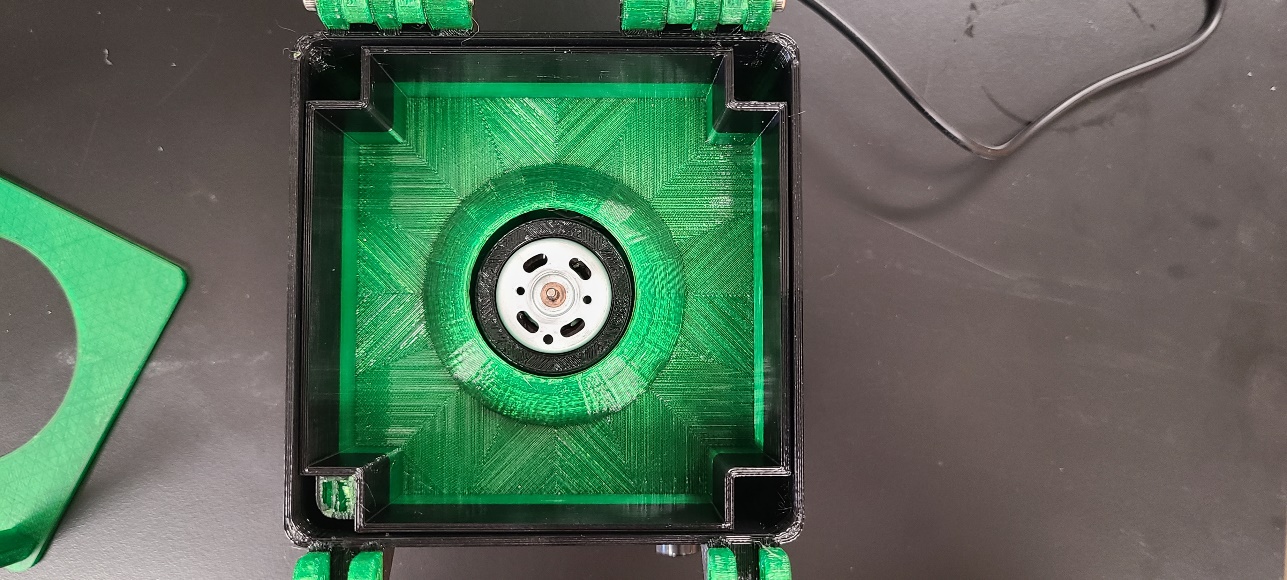


- Insert the shortest Containment Lid Leg into the front left corner of the centrifuge. (It should just sit on top of the micro switch plunger.)
- Using two countersunk M3 bolts, connect the longest Containment Lid Leg to the back left corner of the Containment Lid and the last Containment Leg lid to the corner opposite that.
-
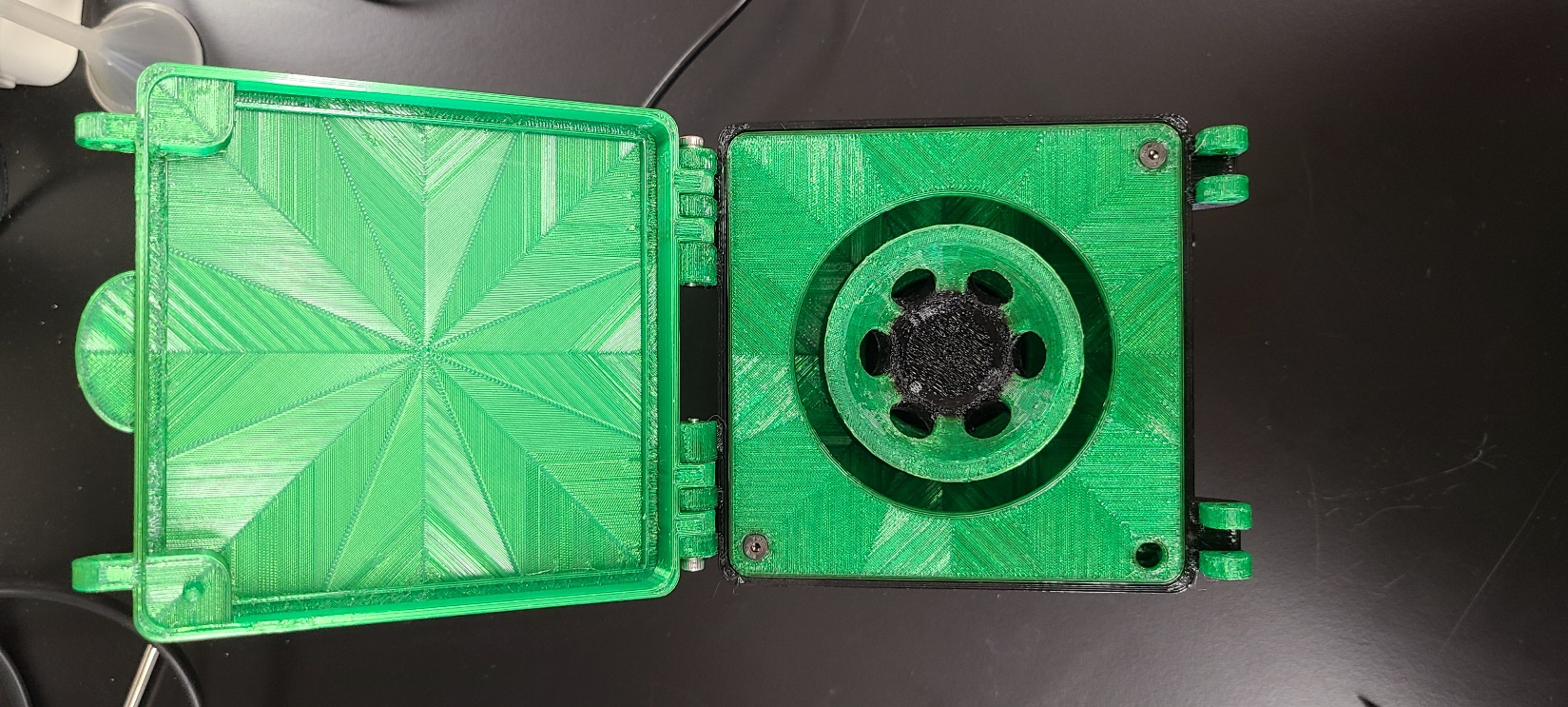
Insert the Containment Lid so the longest Containment Lid Leg inserts into the back left corner of the Centrifuge Body and the small hole counterclockwise from that leg is in the front left corner by the microswitch.
- Insert the Tube Holder onto the motor shaft until it is flush with the containment lid. (It should be a very tight fit.)
-
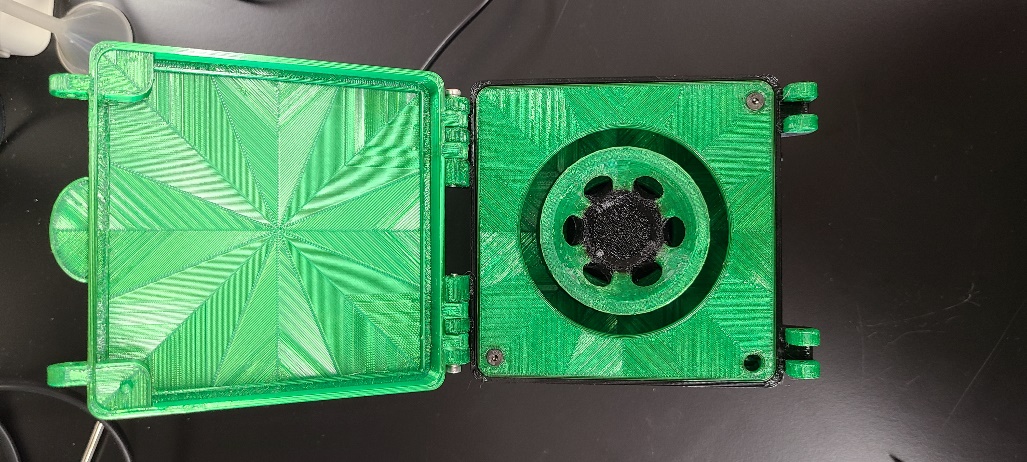
Attach the Centrifuge Lid using two M3 bolts and two hex nuts.
- Close the Centrifuge Lid or push down in the front left corner to activate the centrifuge.
- Adjust the speed using the speed controller knob.

# Sir Tumbalot Tube Tumbler

Bill of Materials

- 3D-printed parts
  - Motor Housing
  - Bearing Housing
  - Rotor
  - 8, 50-mL Tube Modules
- 1, [Pancake Stepper Motor](https://a.co/d/cKmp9Y5)
- 1, [5 x 16 x 5-mm Bearing](https://www.amazon.com/gp/product/B071RXC7FV/ref=ppx_yo_dt_b_search_asin_title?ie=UTF8&psc=1)
- 3, [5-mm x 200-mm Stainless Steel Rod](https://www.amazon.com/gp/product/B00UCR6VOK/ref=ppx_yo_dt_b_search_asin_title?ie=UTF8&psc=1)
- 2, [Flange Coupling Connector](https://www.amazon.com/gp/product/B08334MFVT/ref=ppx_yo_dt_b_search_asin_title?ie=UTF8&psc=1)
- 4, [M3 x 30 Screw Cap](https://www.amazon.com/Team-Associated-91478-Button-3x0-5x30mm/dp/B00J9F9WT2/ref=sr_1_8?content-id=amzn1.sym.918a99dd-4826-4c0a-be33-a6705d69c4cf%3Aamzn1.sym.918a99dd-4826-4c0a-be33-a6705d69c4cf&keywords=Screws&pd_rd_r=950d5165-3eb6-4838-907b-8c7a10dc4584&pd_rd_w=Xeoxd&pd_rd_wg=7vqX8&pf_rd_p=918a99dd-4826-4c0a-be33-a6705d69c4cf&pf_rd_r=TPHFE0JQ4K829N159D7D&pid=2cW0gyP&qid=1677256734&refinements=p_n_feature_five_browse-bin%3A3177279011%2Cp_n_feature_fourteen_browse-bin%3A11434052011%2Cp_n_feature_twenty-eight_browse-bin%3A19043911011&s=industrial&sprefix=M3x30%2Cindustrial%2C70&sr=1-8)
- 16, [12 x 6 x 3-mm Magnets](https://www.amazon.com/gp/product/B09L47HV7M/ref=ppx_yo_dt_b_search_asin_title?ie=UTF8&psc=1)
- 1, [5-mm Shaft Coupler](https://www.amazon.com/dp/B0BP87Q4PC/ref=cm_sw_r_apanp_jQ36YUpTExQSn)
- 1, [Trinamic 2209 Stealth Chop Dev Board](https://www.mouser.com/ProductDetail/Trinamic/TMC2209-BOB?qs=TiOZkKH1s2Qe%2F0oCs8WMDg%3D%3D)
- 1, [Crimp-On Pin Header Kit](https://a.co/d/7HGjm6S)
- 1, [Female Pin Header Kit](https://a.co/d/0HIC8a4)
- 1, [Rubber Feet Set](https://a.co/d/ahQm9Wy)
- Arduino Uno (https://www.arduino.cc/) from various suppliers
- 9-V power supply from various suppliers for Arduino Uno boards
- 22-gauge stranded core wire
- Metric Allen wrench set
- Needle nose pliers

Instructions

- Download and install the TMCStepper Arduino library, linked here: <https://github.com/teemuatlut/TMCStepper>
- Print all the included parts and/or modify the parts in TinkerCAD as linked above.
- Print eight matching tube adapters or design your own to fit that magnetic slot dimension.
- Install 12 x 6 x 3-mm magnets in the rotor, keeping identical polarities facing upward across all eight magnet slots. Use black silicone sealant to adhere the magnets if friction fit is not enough.
- Install similar magnets to each of the tube adapters, check the polarity such that each tube adapter is attracted to the rotor slot magnets. Only test magnetic pull once rotor magnet sealant is cured to avoid ripping magnets out of their slots. Ensure the tube adapter magnets are flush with the flat rectangular surface. Connect all tube adapters to the rotor to check polarities and ensure all tube adapters mate in the same direction.
- Strip 4 mm off each of the wires attached to the stepper motor.
- Crimp on male DuPont connector to each wire and slide on black plastic DuPont connector sheath.
- Solder female pin header rows to the Stealth Chop Dev Board.
- Slide the stepper motor wires through the printed stepper housing.
- Connect the stepper wires to the Dev Board as follows: A1 = Black, A2 = Green, B1 = Red, B2 = Blue.
- Connect the Vio pin to Arduino 3 V.
- Connect the Vmot pin to Arduino 5 V.
- Connect the DIR, STEP, and ENABLE pins on the Stealth Chop Dev Board to Arduino pins 4, 5, and 6, respectively.
- Connect GNDmot and GNDio grounds to Arduino GND pins.
- Connect the motor shaft to the 5-mm rod via the shaft coupler. Ensure the shafts meet in the middle of the coupler and tighten.
- Slot the 5-mm bearing into the printed bearing housing.
- Slot the bottom 5-mm rods to the housing feet and push everything together, aligning the 5-mm motor rod into the 5-mm bearing bore.
- Upload the included code to the Arduino; the motor should begin rotating. If it makes a stall noise, just give the motor rod a manual twist in the direction of rotation to overcome initial friction. Take note of any wobble or eccentricities. If there is wobble, cut power to the Arduino and adjust the shaft coupling as that is the central source of wobble. There will always be a bit of wobble due to shaft manufacturing defects.
- Attach the two flange couplers to the rotor and fasten with M3 x 30-mm screws. Be mindful of the tiny worm screws in the flange as they may get lost.
- Disassemble the bearing housing from the 5-mm rods and slide the rotor onto the 5-mm motor shaft, center it, reattach the bearing housing to all the rods. Once the rotor is centered, screw in the worm screws to secure the rotor to the motor shaft.
- Ensure everything is pushed together firmly. Apply rubber feet to the bottom of each printed housing. Turn on the Arduino and see if it spins. Remember, if it stalls, just give it a twist to help it start. The low torque is a safety feature unintentionally designed but very much warranted given the nature of the device. Low torque implies far less of a chance of bodily or equipment harm in the event of a stall due to a snag on objects or clothing.

# Additional 3D Printing Resources

We highly suggest checking All3DPrint.com (All3DP, 2023) or the pinned post in r/3Dprinting on Reddit for in depth buying guides (/u/richie225, 2023).

Troubleshooting and printer calibration and optimization guides are numerous but Simplify3D and r/FixMyPrint on Reddit both provide great resources (Simplify 3D, 2023; U/madeinchina et al., 2022).

There are also multiple active communities on discord *(Creality, 2023; Voron Design, 2023; Print Everything Discord,* 2023; Prusa3D, 2023).

All3DP. 2023. Best 3D Printing Services in 2023 – Buyer’s Guide. All3DP. Internet Archive Website: <https://web.archive.org/web/20230709103428/https://all3dp.com/1/best-online-3d-printing-service-3d-print-services/> [accessed 5 December 2023].

Creality. 2023. Creality 3D Printers Official Discord. Internet Archive Website: <https://web.archive.org/web/20221121011834/https://discord.com/invite/Ay3sBqXAG7> [accessed 5 December 2023].

Polymaker. 2023. Polymaker Discord. Internet Archive Website: <https://web.archive.org/web/20221130042510/https://discord.com/invite/polymaker> [accessed 5 December 2023].

Print Everything Discord. 2023. Internet Archive Website: <https://web.archive.org/web/20220614164520/https://discord.com/invite/DzZmRms8j7> [accessed 5 December 2023].

Prusa3D. 2023. Prusa3d Discord. Internet Archive Website: <https://web.archive.org/web/20220614164520/https://discord.com/invite/cjk3FuJ> [accessed 5 December 2023].

Simplify 3D. 2023. Print Quality Guide. Simplify 3D [online]. Internet Archive Website: <https://web.archive.org/web/20230702191939/https://www.simplify3d.com/resources/print-quality-troubleshooting/> [accessed 5 December 2023].

U/madeinchina, U/toddthefrog, and U/scuffling. 2022. r/FixMyPrint Wiki. Internet Archive Website : <https://web.archive.org/web/20220623190748/https://www.reddit.com/r/FixMyPrint/wiki/index> [accessed 5 December 2023].

/u/richie225. 2023. Generic FDM Printer Recommendations, 2022: 3Dprinting. Reddit r/3Dprinting. Internet Archive Website: [https://web.archive.org/web/20230405131842/https://www.reddit.com/user/richie225/comments/rnillw/generic_fdm_printer_recommendations_2022/](https://web.archive.org/web/20230405131842/https:/www.reddit.com/user/richie225/comments/rnillw/generic_fdm_printer_recommendations_2022/) [accessed 7 June 2023].

Voron Design. 2023. Voron Discord. Internet Archive Website: <https://web.archive.org/web/20230305022133/https://discord.com/invite/voron> [accessed 5 December 2023].
